# Mortality inequalities in France since the 1920s: Evidence of a reversal of the income gradient in mortality

**DOI:** 10.1371/journal.pone.0280272

**Published:** 2023-01-17

**Authors:** Florian Bonnet, Hippolyte d’Albis, Josselin Thuilliez

**Affiliations:** 1 INED (Institut National d’Etudes Démographiques), Aubervilliers, France; 2 Paris School of Economics, Paris, France; 3 CNRS (Centre National de la Recherche Scientifique), Centre d’économie de la Sorbonne, Paris, France; Max Planck Institute for the Science of Human History, Jena, Germany, HUNGARY

## Abstract

Many recent studies show that Europe has had a lower mortality inequality for most ages than the United States over the last thirty years. However, the evolution of the income gradient in mortality all along the twentieth century remains poorly understood. This article uses a unique dataset that gives the annual lifetables and fiscal income for the 90 administrative regions of mainland France from 1922 to 2020. The income gradients in mortality are computed across regions using a traditional method with calendar ages and, alternatively, with mortality milestones to control for the increase in life expectancy over time. The study reveals a systematic reversal of the gradient that occurred around the 1970s for both sexes and all ages or mortality groups when calculated at an aggregated level. Inequality in mortality amongst the oldest age groups has however returned to a level observed at least ten years earlier because of Covid-19, even after controlling for mortality improvements over the period.

## Introduction

Mortality inequalities across socio-economic groups are often measured with the income gradient in mortality. A positive gradient means that economic inequalities are more harmful to disadvantaged populations, since economically unequal lives are associated with unequal lifetimes, and are consequently riskier for a society. Over the last decades, the income gradient in mortality has increased for some populations. This has been, most notably, established for adults in the US using individual [1] or county-level data [2, 3], while the picture was more optimistic for the youth [4, 5]. Trends in inequalities in mortality have been more favorable in other high-income countries [6, 7] and, in particular, in Canada [8] or France [9]. In the latter, inequality in mortality remained remarkably low and stable from 1990 to 2018. These differences across ages and countries question, among others, the ability of health and social protection systems to prevent economic inequalities from translating into health inequalities [10].

The factors that influence the gradient are very diverse, ranging from the individual to the macro scale [11]. Individual factors are typically income, social position, or education, while aggregate factors cover urbanization, environment, or medical supply and social coverage. Moreover, it is established in the economic literature that results can depend on the scale of analysis [12–15]. Our paper considers the relationship between income and mortality using relatively large regions; it is therefore at the level of aggregate factors and does not allow an analysis of individual dynamics. It is based on a series of articlesthat used to characterize inequalities in France and the United States [4, 16], and has the advantage of allowing precise computation of mortality rates, particularly for ages where mortality is low, and for compositional changes (e.g. selective migration) over time. The method is applicable for multi countries or single-country analysis over long periods of time. On the other hand, this method does not allow us to characterize inequalities between individuals and propose causal analyses.

Despite the abundant empirical literature evaluating the income gradient in mortality in high-income countries, historical perspectives have been scarce and most studies present evaluations that start in the 1990s. Several studies also focused on the individual level. This lack of historical evidence is challenging since the analysis over the long run can provide new insight into social dynamics, as exemplified by the research on economic inequalities [17]. Among the rare exceptions is [18], who used Norwegian data since 1960 and show that mortality inequality across educational groups widened between the 1960s and the 1990s. [19–21] pointed out that the social class pattern of mortality for men in England and Wales, particularly coronary heart disease-related deaths, seemed to be changing from the 1950s onwards. Moreover, [22] studied mortality conditions in two provinces of the Netherlands between 1820 and 1929. Interestingly, they suggested that the gradient was larger in the early twentieth century and has declined since then. Conversely, in an impressive study covering Southern Sweden between 1813 and 2015, [23] have recently shown that a positive gradient for adult men emerged only after 1970 and was flat before that. Using the 1960 census, [24] also examine mortality by causes and social groups in Sweden but do not say much about the evolution of the gradient over time. They show that differences in mortality between socio-economic groups may be explained partly by changing smoking habits, age, gender, urbanization, region of residence and marital status. This conflicting evidence calls for further investigations.

We offer a historical approach based on French regional statistics by using a unique database that provides annual demographic and economic indicators for 90 administrative areas of mainland France from 1922 to 2020. An analysis of France’s income gradient over a century can be viewed as a comparison between different countries, as the context has changed so much. Therefore, we start our analysis with methods used for cross-country comparisons [25]. For each income group and each year, mortality measures are computed. Our results are presented in a range of graphs. We first follow the literature and compute the average absolute death rates by income group, with further decomposition by sex and by age group. This enables us to analyze trends across ages and gender in places with the same relative income. Factoring in age is important to account for whether changes in life expectancy are based on a person’s stage of life. Our graphs reveal a general reversal of the gradient that occurred around the 1970s for both sexes and all ages or mortality groups. The gradient was thus negative before the 1950s, a result which has not been established yet in the literature.

We next complement these results with an original standardization method that permits to control for the historical improvement of longevity. In 1920, a 20-year-old French man on average had a remaining life expectancy of 43 years, against 60 years today; in fact, 43 years corresponds to today’s life expectancy for a 38-year-old man. It is therefore more relevant to compare over long time populations who face the same mortality conditions rather than are the same age. Using remaining life expectancies to define a “prospective age” rather than chronological ages was found to be suitable to compare populations over a long period or across space and sexes.

The novelty here is to build on this idea and to control for mortality improvements when providing a historical analysis of the income gradient. Rather than using age to define the relevant population, we thus use their annual death rate. This standardization strategy is based on the concept of mortality milestones [26], which consists in selecting ages at which men and women reach certain thresholds in terms of risk of dying during the year. By using death rates, this population grouping is similar, while simpler to implement, to what would be obtained with remaining life expectancies as it integrates mortality improvements. The main drawback is that we exclude infant mortality and focus on ages for which mortality increases with age. With this standardization, our earlier results on the reversal of the gradient appear even more clearly.

The income gradient in mortality, which was negative one century ago, turned positive in the 1970s. Some rich regions used to display relatively high mortality patterns, especially in the industrialized North-east of France, while today, the average mortality conditions are relatively better in rich regions. Interestingly, the reversal of the gradient occurred while inequalities across regions continuously reduced for both mortality [27] and income [28]. Our results shed some light on the ongoing debate on mortality inequalities and their relations to economic development and inequalities. In an influential paper, [10] suggested that there was no direct link and concluded that stories about income inequality affecting health were “stronger than the evidence”. Since then, many authors have highlighted the impact of economic inequalities on health insisting, in particular, on the psychological effects induced by competition among individuals [11].

Using a large dataset for the US, [1] confirmed that income inequalities are associated with longevity inequalities, but the literature is still non conclusive about causal effects [29]. Within these studies, geographical differences in longevity are often mentioned and more and more analyzed. In particular, [30] insist on the roles of the health care system, climate and socioeconomic status. We complement them by offering a long-run perspective and highlight the mortality history in rich regions. They used to exhibit higher average mortality rates while they are now those with the lowest mortality levels. This finding echoes a literature exhibiting a so-called “urban penalty”, describing excess mortality in large cities during the 19^th^ century [31]. Here, the story is different since excess mortality is found to concern industrialized regions and last till WWII.

The rest of the article is structured as follows. We first detail the data and the methodology we use. Second, we present our main result using a standard computation of the gradient. Third, we compute again this gradient using mortality milestones. Fourth, we present the evolution of the gradient with that of the spatial distribution of income in France. Finally, we propose a discussion and conclude.

### Data

We use a unique database that provides annual demographic and economic indicators for the 90 administrative regions of mainland France from 1922 to 2020. This database merges and extends two recent databases.

In the first one, [32] uses civilian deaths, military deaths, and births by year as well as population by age at census dates to reconstitutes annual life tables for each French NUTS3 regions (named *département*s in French) and both sexes from 1901 to 2014. The methods used to reconstitute these life tables are very similar to those used to reconstitute the national life tables of countries available in the Human Mortality Database. For this article, we use deaths and populations by age at the regional level for the period 1922–2014. Moreover, we have extended this database to the years 2015–2020 using deaths by single age and populations by age groups available from INSEE. For these specific years only, we are forced to consider the population at age 0 as the births of the year, and the population at ages 1 to 4 as the population at ages 0 to 4 minus births of the year. The age-specific mortality rates are obtained by dividing the total number of deaths by the average population of the year.

In the second one, [28] rely on [33] and aggregate statistics about income tax to reconstitute fiscal incomes for the same regions since 1922. In a first step, [33] use detailed statistics about income tax (number of tax units and level of income declared by brackets of income) to reconstitute annual distributions of fiscal income for each French *département*s from 1960 to 1969 and from 1986 to 2015. The methods used to reconstitute these distributions of fiscal income are very similar to those used by [34] to reconstitute the French national distributions of fiscal income. For this article, we have extended this database to the years 2016–2020 following the same methodology. In a second step, [28] use both population age structure and aggregate statistics about income tax (number of imposable tax units, income tax paid by imposable tax units, income declared by imposable tax units) to calibrate a model on years 1960 to 1969 and 1986 to 2015. They use this model to estimate fiscal income for each French *département*s from 1922 to 1959 and from 1970 to 1985. For this article, we use fiscal incomes for the period 1922–2020 (fiscal incomes without capital gains). Fiscal income is defined as the sum of (1) labor income after payment of both employer and social contributions and (2) capital income after payment of social contributions; it considers pension benefits but not social benefits. The per capita fiscal incomes used are obtained by dividing the total fiscal income by the number of inhabitants (S1 and S2 Tables in S1 Appendix for descriptive statistics).

Note that in 1968, the 3 *départements* of the Paris region were split into 8 *départements*. We kept the classification that existed until 1967 by summing the deaths and populations of the new *départements*. We show below that this change in classification does not affect the results. A map of the 90 administrative regions we consider is given in the S0 Fig in S1 Appendix.

## Methods

We follow [4] and create 20 groups of regions, which are ranked according to their average income. Groups, which have the same population size, are created from an abstract population that assumes that each inhabitant of a given region receives the regional average income. Group 1 refers to the top 5th percentile of the distribution of this abstract population, while group 20 is the bottom 5th percentile. This grouping is made for all years of the period considered, which may imply some reassignments of regions from one income group to another to account for changes in regions’ relative population and income. Due to a shorter time window, [4] show that keeping groups (of US counties, in their case) invariant does not change their results; in our case, due to a much longer time window, those reassignments are crucial. To quantify the relationship between per capita income and age-specific mortality rates, we use a least squares regression as represented in Eq (1):

$$m_{vt}=\alpha +\beta *RI_{vt}+\varepsilon_{vt},$$ (1)

where *v* represents the ventile and *t* the year, *q* is the mortality rate, *RI* the rank of income per capita. *ε*_*vt*_ is the error term. The coefficient β represents the mortality gradient according to income rank. It reflects the difference in mortality levels between two successive ventiles. A zero value means that there is no mortality inequality according to per capita income rank. A positive value means that mortality is higher and income per capita is lower. Note that to check for the robustness of our results, we also estimate the mortality gradient according to the value of income per capita rather than the rank using the following equation:

$$m_{vt}=\alpha +\beta *I_{vt}+\varepsilon_{vt},$$ (2)

where *I* is the income per capita.

### The reversal of the income gradients in mortality

**Fig 1:**
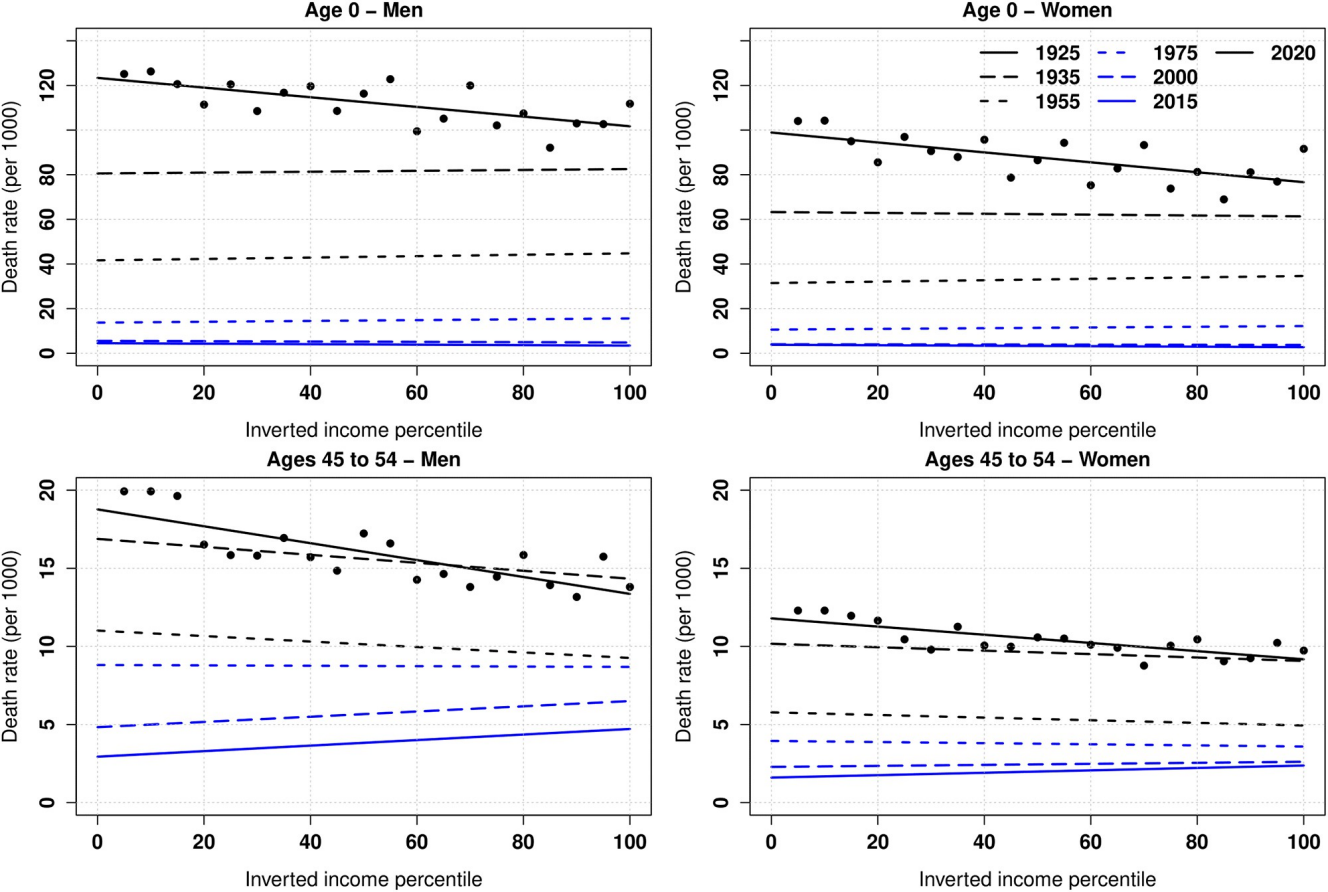
Death rates by inverted income per capita percentile. Lines represent the linear trend that fits the annual death rates per 1,000 for selected ages and sex across 20 income groups. A positive slope indicates that mortality decreases with income. In the S1 Appendix, figures for all age groups are provided for both men (S1 Fig in S1 Appendix) and women (S2 Fig in S1 Appendix).

For each income group and each year, mortality measures are computed. Our results are presented in a range of graphs. We first follow the literature and compute the average absolute death rates by income group, with further decomposition by sex and by age group. Fig 1 presents a selection of the gradients for men (left panels) and women (right panels) at ages 0 (upper panels) and 45–54 (lower panels) by inverted income ranking: we represent the estimates for each income group with a dot for years 1925 and 2015, and the linear trend fitting those estimates for the same years and years 1935, 1955, 2000, and 2015. Using an inverted income ranking permits interpreting a positive gradient as a mortality that decreases with income.

Two main observations can be deduced from Fig 1. First, it illustrates the strong decrease in mortality that all age groups and all income percentiles experienced over a century. For instance, infant mortality for men declined from 105 per thousand in 1925 to 3.5 per thousand in 2015. Second, the gradient appears to be strongly negative at the beginning of the period and to be flat (for younger ages) or positive (for older ages) at the end of the period. For the last decades, our results are consistent with the literature [16, 25]. This analysis was redone by distinguishing men and women and for eleven age groups (S1 and S2 Figs in S1 Appendix), which confirmed the main results. Note that if we compute the gradients as functions of the absolute income (see Eq 2), the results are similar but the graphical representations are less readable (S3 Fig in S1 Appendix).

**Fig 2:**
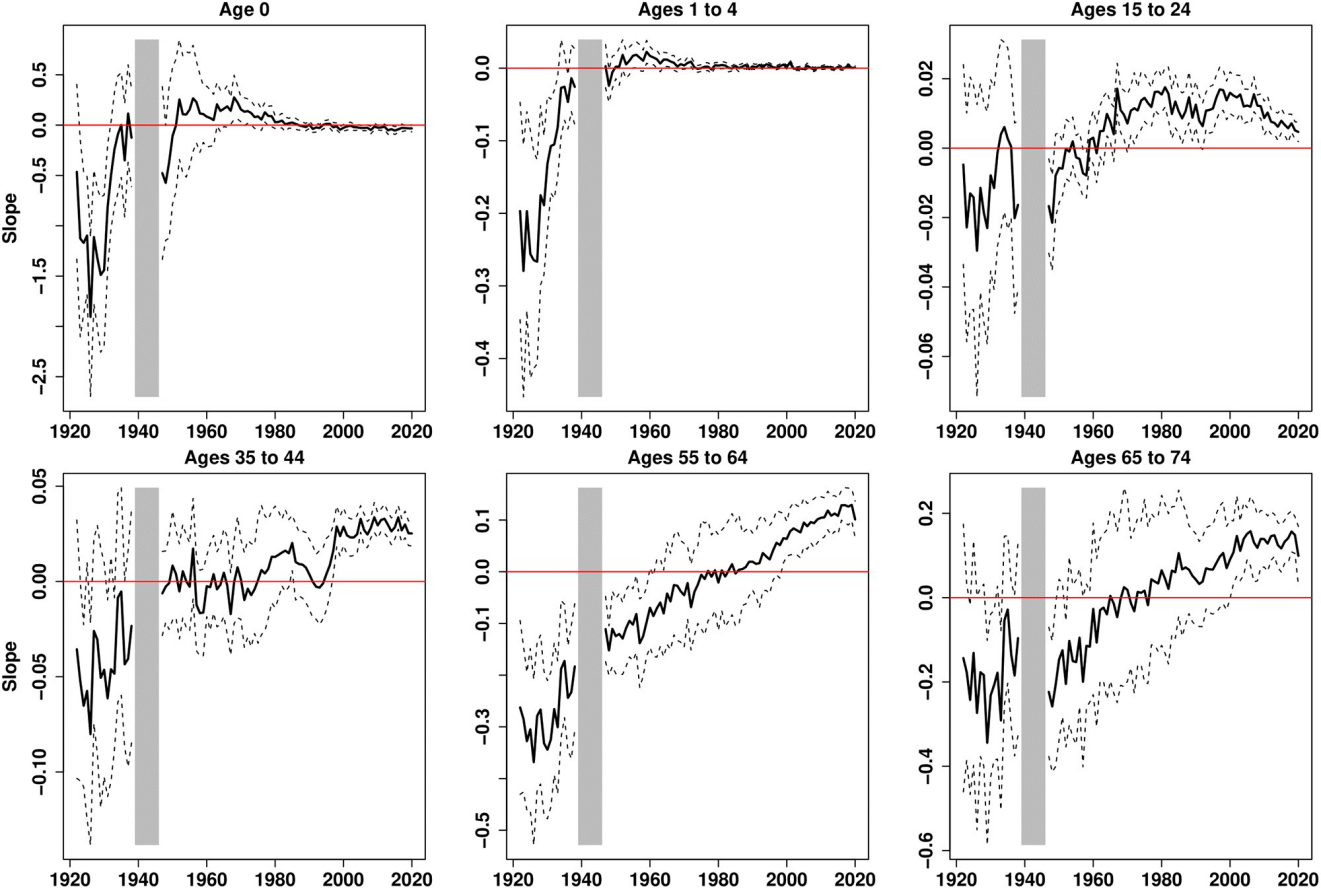
Income gradient in mortality for 6 age groups, 1922–2020. Plain curves plot the estimates of the linear trends across income groups; dotted lines are the 95% confidence intervals. Gradients are not represented for the period 1939–1945.

Fig 2 systematizes this first analysis for all years by representing the evolution of the income gradient in mortality, which is given by the slope of the lines represented in Fig 1, and the 95% confidence interval associated with its estimation. Income gradients for a selection of six age groups, without distinguishing between the sexes, are represented here. For infants, the gradients are significantly negative until the 1930s and are null from the 1970s onward. For young adults, the gradient is not significantly different from zero at the beginning of the period and becomes positive in the 1960s for ages 15–24 and in the 2000s for ages 35–44. For older adults, the gradient is initially significantly negative and ultimately significantly positive. We notice a significant decline of the gradient after age 55 in 2020, which is due to the high prevalence of SARS-CoV-2 in the richest regions, and in the Paris region in particular, during the first wave of the pandemics.

This analysis is replicated for men and women and eleven age groups (see S4 and S5 Figs in S1 Appendix), which confirms the main results. A significantly negative gradient is observed for younger ages before 1935, while the gradient is significantly positive for older ages after 2000. Whatever the sex or age of the considered population, the gradient is generally increasing over time. The main exception is observed for ages 25–34, for which the gradient was significantly positive in the 1930s, possibly due to selective migration issues: healthy young adults may have been attracted by rich regions for work. For ages 85+, the reduction of the gradient in 2020 is impressive. In 1968, the 3 *départments* of the Paris region were split into 8 *départments*. We computed the gradients with this new classification from 1968 onward and did not find significant changes in the evolutions except that there is no decline in 2020 for the 65–74 years old (S6 Fig in S1 Appendix). This is explained by the fact that the highest excess mortality during the pandemic mainly affected the poorest *départment* in the Paris region, namely the Seine-Saint-Denis.

### Computing the gradients using mortality milestones

The above results could be critiqued due to the use of chronological ages to analyze the evolution of populations’ health conditions over a long period. It may indeed seem curious to compare income gradients for a given age group over time since life expectancies have drastically increased. In 1922, a 20-year-old French man on average had a remaining life expectancy of 43 years, against 60 years today; in fact, 43 years corresponds to today’s life expectancy for a 38-year-old man. It is therefore more relevant to compare over time populations who face the same mortality conditions rather than are the same age. Using remaining life expectancies to define a “prospective age” rather than chronological ages was found to be suitable to compare populations over long periods or across space or even across sexes [35, 36].

The novelty here is to follow this idea and to control for mortality improvements when providing a historical analysis of the income gradient. Rather than using age to define the relevant population, we thus use their annual death rate. This standardization strategy is based on the concept of mortality milestones, proposed by [26]. The strategy is to consider the ages at which individuals reaches a given threshold in terms of the risk of dying during the year. For each year, we select the age at which the threshold is reached in average in France, and then use this age to compute the gradient. By using death rates, this population grouping is similar, while simpler to implement, to what would be obtained with remaining life expectancies as it integrates mortality improvements. The main drawback is that we do not look at infant mortality and rather focus on ages for which mortality increases with age.

**Fig 3:**
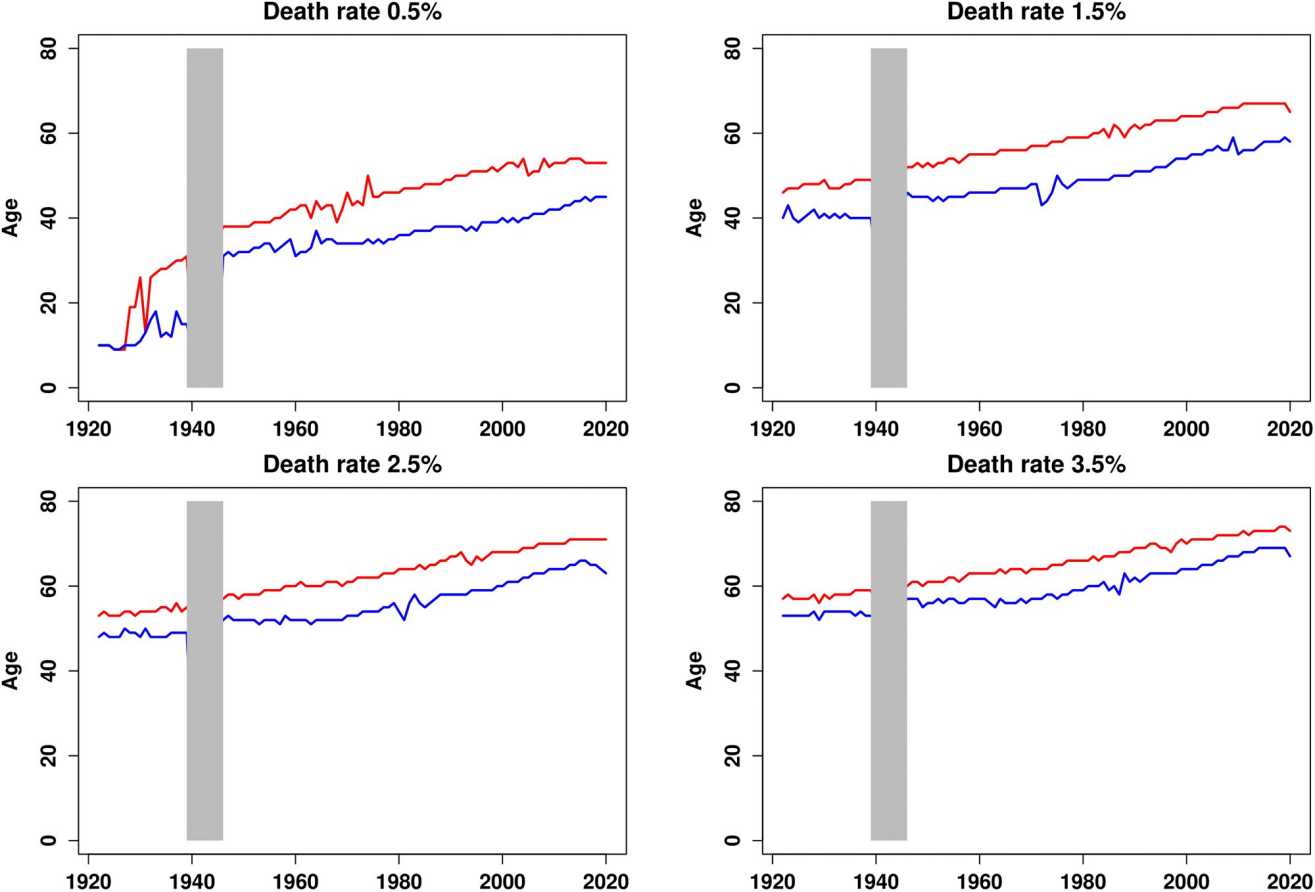
Ages at which 4 mortality milestones are reached, 1922–2020. Plain curves plot the ages that correspond to the mortality threshold considered. Men are in red and women are in blue. Ages are not represented for the period 1939–1945.

Fig 3 represents the ages at which various mortality milestones, corresponding to annual death rates of 0.5, 1.5, 2.5, and 3.5 per thousand, are reached over time. There are monotonic increases for most death rates and both sexes. For instance, men in the 1930s faced a death rate of 2.5 per thousand before age 50, while today the same death rate is reached at age 65. The evolution of the sex gap in death rates closely follows the well-known evolution of the sex gap in life expectancy. This difference increased sharply after WWII due to a high smoking prevalence among men, which increased their avoidable mortality, in contrast to women. Thereafter, this gap stabilized, before starting to decrease at the end of the 20th century with the progressive decline in smoking among men and its opposite development among women.

**Fig 4:**
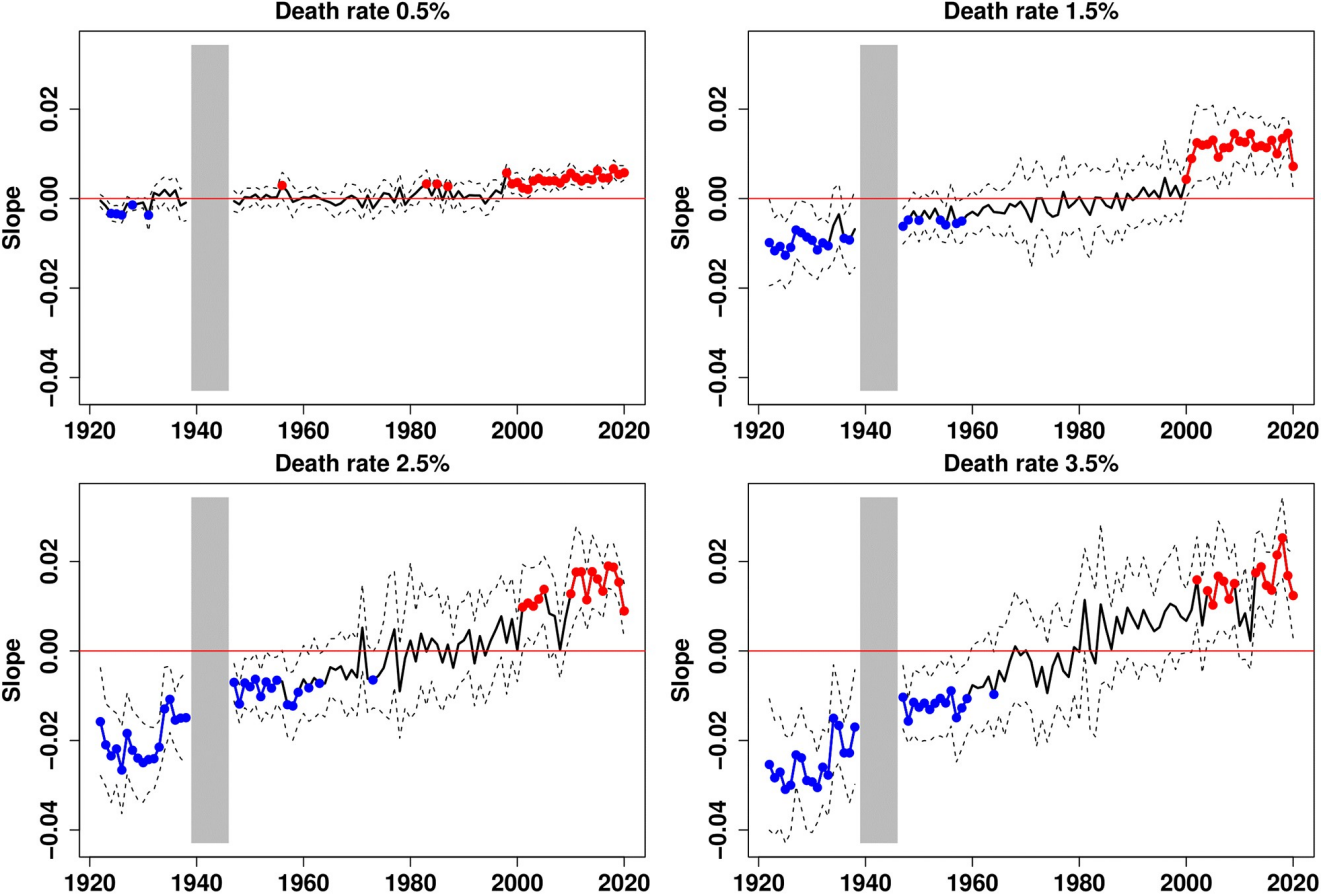
Income gradient in mortality for 4 mortality milestones, 1922–2020. Plain curves plot the estimates of the linear trends across income groups; dotted lines are the 95% confidence intervals. Red dots indicate dates at which the slope is significantly positive, while blue dots indicate those with significantly negative slopes. Gradients are not represented for the period 1939–1945.

Then, the income gradients can be calculated as in Eq 1. However, instead of taking fixed ages for each year, we take the mortality milestones as the reference. The results are provided in Fig 4. The historical trend of the gradients is now clearly positive and the reversal of the income gradient from negative to positive values now significantly occurs for all mortality milestones larger than 1 per thousand. Importantly, the evolution of the gradient is much flatter for the lower death rates and the reversal is more pronounced for higher ones. The period during which the gradient is not significantly different from zero reduces as mortality rates increase. It ranges from the late 1940s to the late 1990s for a death rate of 0.5 per thousand, and from the early 1970s to the late 1990s for a death rate of 3.5 per thousand. The 1970s appear to be a turning point that marks the reversal of the income gradient.

The analysis presented in Fig 4 was replicated for men (S7 Fig in S1 Appendix) and women (S8 Fig in S1 Appendix). The general picture remains valid, except at the beginning of the period considered for men facing a 1.5 per thousand death rate who barely experienced a negative gradient and for women facing a 0.5 per thousand death rate who experienced a positive gradient. Interestingly, the evolution is generally much flatter for women than for men. We also recomputed the gradient by excluding the three most densely populated regions of France (S9 Fig in S1 Appendix), i.e. by excluding the regions of the three largest cities, Paris, Lyon, and Marseille. Again, the results are qualitatively similar, and we can see that our results are only slightly affected for the first half of the 20th century but not during the second half of the twentieth century.

### The gradient and the transformation of the geography of wealth

**Fig 5:**
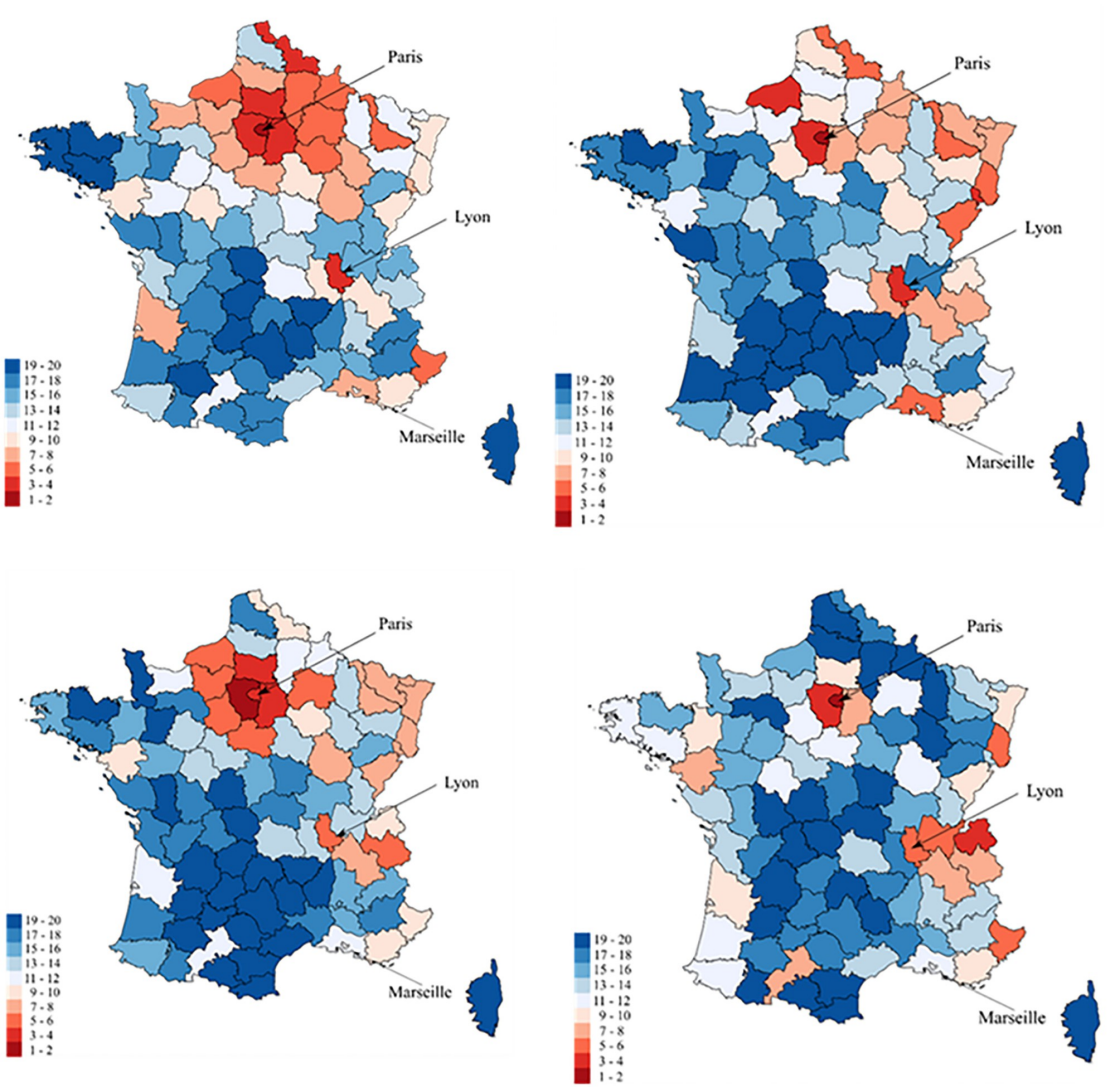
20 ventiles of départements in 1925, 1950, 1975 and 2020. Départements are classified according to their place in the 20 ventiles. The richest départements are in dark red while the poorest ones are in dark blue.

To calculate the gradients, we form each year twenty groups of *départements*, ordered by their average income. During the hundred years that we are considering, there are naturally *départements* that have moved between groups. Fig 5 shows some of these modifications by coloring the *départements* by pairs of ventiles.

**Fig 6:**
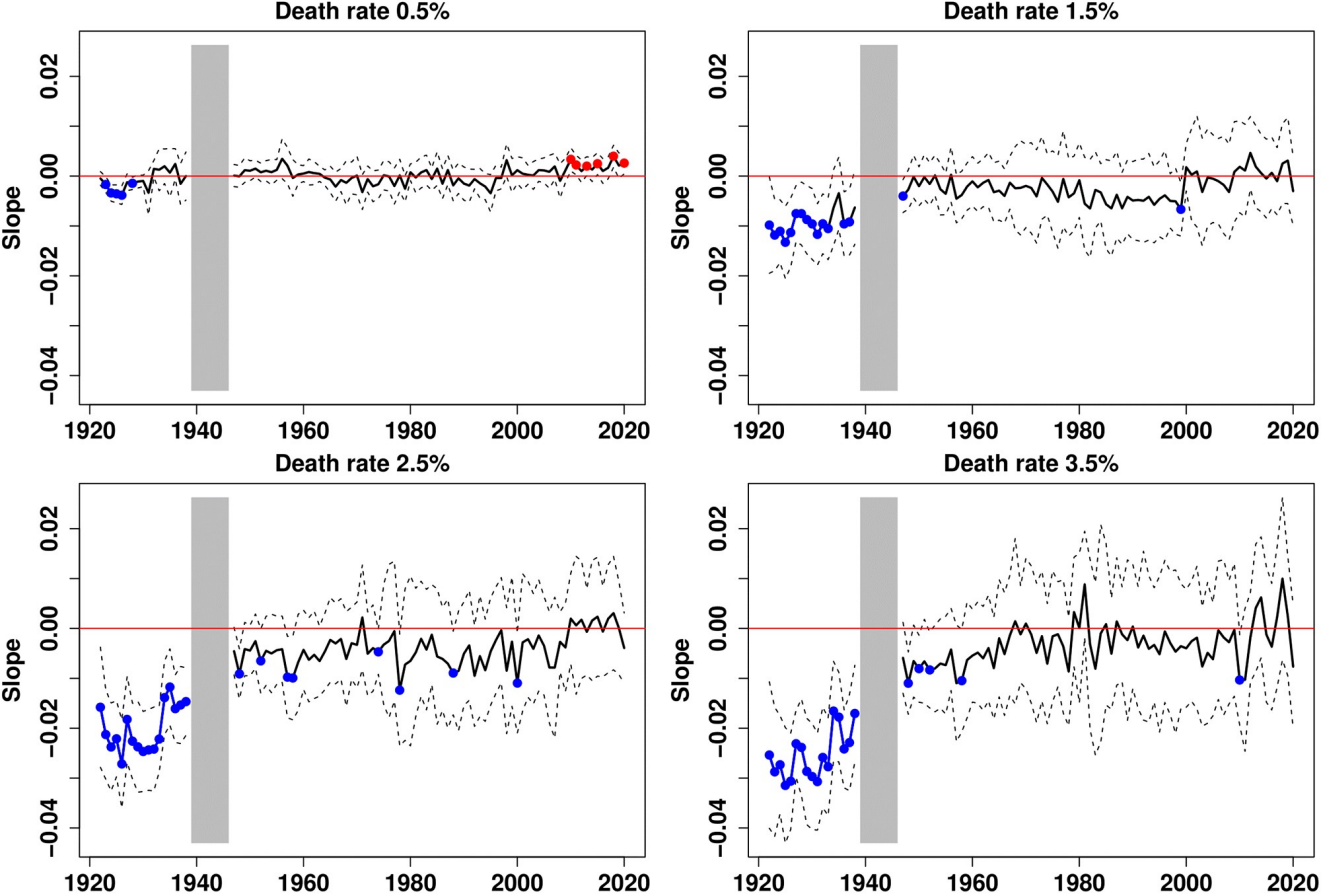
Income gradient in mortality for 4 mortality milestones, 1922–2020 (1922 classification). Plain curves plot the estimates of the linear trends across income groups; dotted lines are the 95% confidence intervals. Red dots indicate dates at which the slope is significantly positive, while blue dots indicate those with significantly negative slopes. Gradients are not represented for the period 1939–1945.

Fig 5 reveals that the departments of Paris and Lyon and some other regional capitals, as well as those of the South-East have systematically been among the richest in France. Conversely, the departments of the once-industrial regions of the North-East experienced an impressive relative decline. They were among the richest until WWII and are now among the poorest. To assess the effect of the transformation of income spatial inequalities on the gradients we computed, the groups of *départements* can be kept fixed throughout the period considered. In Fig 6, the 1922 classification is kept constant. The evolution of the gradients changes radically, whether they are calculated using mortality milestones, or in the standard way (S10 Fig in S1 Appendix). In most cases, the gradient is no longer significantly positive at the end of the period. The northeastern regions are (artificially) kept among the richest, and because of their relatively higher mortality, the positive gradient is lost.

**Fig 7:**
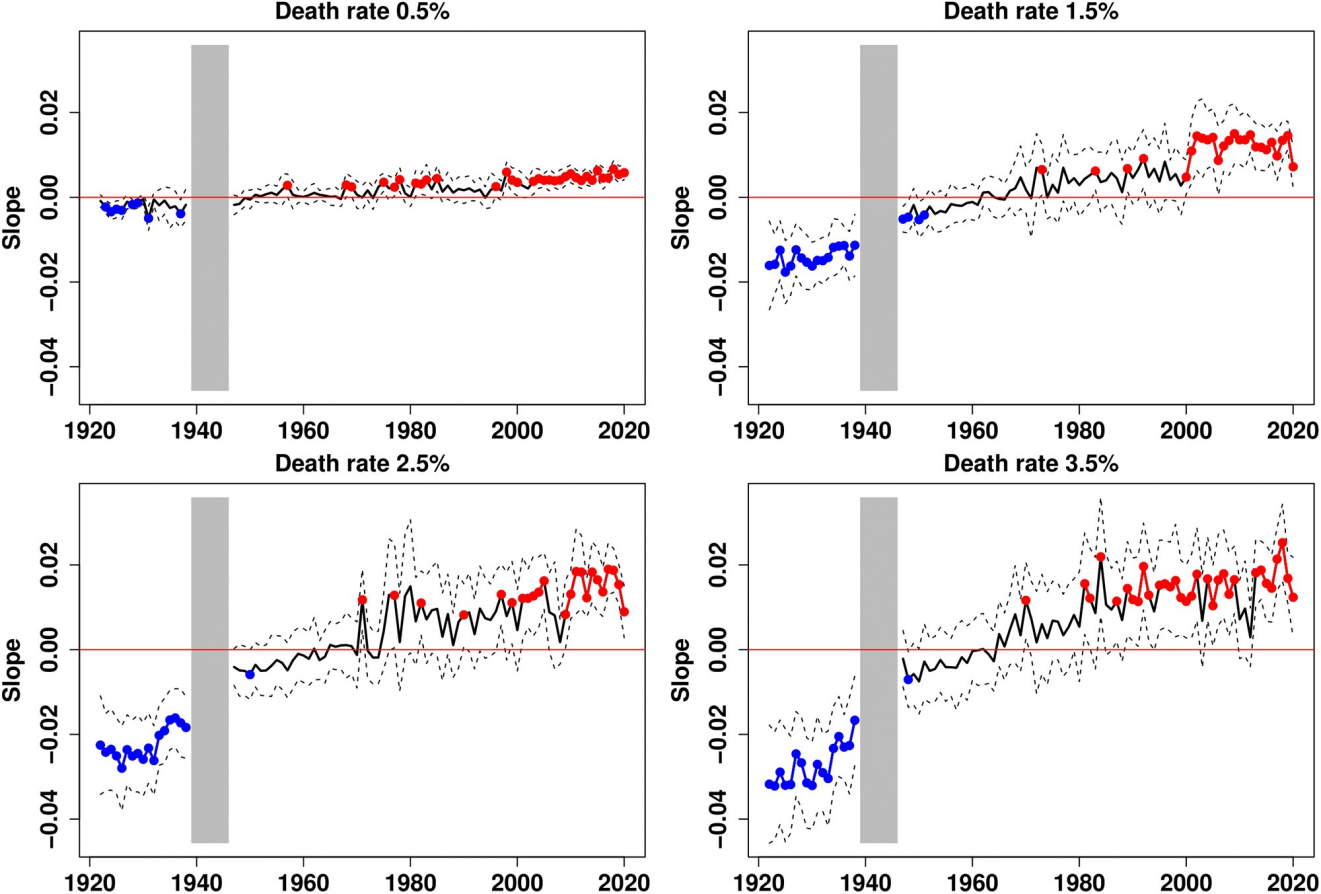
Income gradient in mortality for 4 mortality milestones, 1922–2020 (1922 classification). Plain curves plot the estimates of the linear trends across income groups; dotted lines are the 95% confidence intervals. Red dots indicate dates at which the slope is significantly positive, while blue dots indicate those with significantly negative slopes. Gradients are not represented for the period 1939–1945.

Conversely, by keeping the classification of 2020 constant throughout the period, we observe that the reversal of the gradient occurs earlier. Fig 7 indeed shows that the gradient is practically no longer negative after 1945 and that it becomes positive in the 1960s (see also S11 Fig in S1 Appendix). In 2020, the wealthiest regions are those with the largest cities. Since health conditions in these areas have greatly improved over the course of the century [27], the gradient reversed more quickly.

## Discussion and conclusion

The reversal of the income gradient in mortality echoes the findings of the literature on the “urban penalty” [31] which describes the comparatively high death rate of densely populated rich European cities in the nineteenth century [37–40], mostly due to their poor sanitary systems in times when the prevalence of epidemic diseases was high. This urban penalty became an “urban advantage” during the 20^th^ century, as [27] revealed for France. Note also that using data from 2010 to 2016 on metropolitan areas of the Americas, [41] show that more populated cities have lower mortality in U.S. cities. The urban advantage may be explained by a more educated population and better access to health care facilities in urbanized regions [42, 43]. We complement this literature by showing that the urban penalty (translated here into a negative income gradient) was still at play until the 1960s for specific mortality groups. To reinforce this interpretation, we recalculate our gradients using population densities rather than average income. S12 Fig in S1 Appendix is the counterpart of Fig 2, except that we use population densities instead of mean income. The patterns are similar, especially for the early period.

A tentative interpretation of our findings is the following. The reversal of the income gradient reflects the economic development process which was industry-based until the 1960s, at the cost of poor health conditions for the workers involved and of highly polluted neighboring environments, and has shifted to a service-based economy that preserves the health of its employees and of populations that live close to the main economic centers. This interpretation is consistent with the differential evolution over time observed for the income gradients of men and women: since the participation rate of the latter in the labor market was much lower in the early twentieth century (42.5% in 1921 according to [44]), their gradient was less negative. Three phases of development can therefore be envisaged. Until WWII, the richest regions are those of the largest cities and the industrial regions of the northeast. Working conditions, pollution, low social coverage and the density of cities were detrimental to health. Accordingly, the gradient was negative. After WWII, the productive system changes and social security became widespread. Large cities were benefiting the most from this progress. The gradient became flat. Since the 1970s, the economic dynamic is based on service industries employing high-skilled workers. These sectors are mostly located in large cities that offer a more efficient healthcare system that the rest of the country. The gradient then became positive. These interpretations should of course be rigorously proven, which calls for further research and data collection. Moreover, other interpretations are possible, most notably those relying on cultural habit differences across regions.

Our analysis may hold the potential to initiate new avenues for historical demographic research. To our knowledge, this study cannot be replicated today in another country, which is the originality of the study, but it would be particularly beneficial to have this same type of data in the US for example. Our analysis suggests that both the structural transformation of the economy and the extension of the healthcare system may have favored the wealthier regions by allowing them to catch up and then overtake the leaders. Though these explanations are not causal, it is striking that the situation looked globally more equal in the 1970s, as mortality was not significantly related to income. Inequality in mortality continued to decline after 1970, but the residual differences appear to be increasingly related to the level of development.

To conclude, we may list six potential limitations of our study. First, the use of average incomes for administrative regions ignores potential differences in regions’ income distributions. To date, there is no statistical information available on those distributions for the long period considered. Second, selective migration is also a potential concern, if e.g. the healthiest people leave economically declining areas. This may explain why we find that the gradient of young adults (25 to 34-year-old)—who form a very important component of urban populations—is positive in the beginning of the considered period. This problem is usually addressed by considering fixed groups of regions [45], but -as we discuss above- this method is less relevant over a long period. Third, we do not adjust regional incomes for differences in the cost of living. This would require historical series of regional inflation, which do not exist. Reconstituting them is a major scientific challenge that it would be interesting to address. Fourth, our analysis focuses on mortality trends, and not on other health characteristics. It would be nice to decompose the gradient by causes of death. Fifth, we recognize that our analysis, made of original figures, is descriptive and does not provide any causal statement. Such analysis would require additional data collection, and is left for future research. Finally, we acknowledge the fundamental limitation of this type of aggregative study, which is that they cannot distinguish the role of compositional effects within regions in mediating the relationship between average income and average mortality rates. Regions may differ in the unobserved characteristics of their populations.

## Supporting information

S1 Appendix Its contains all the supporting tables and figures. (DOCX)
