# Supplementary material for: Mortality inequalities in France since the 1920s: Evidence of a reversal of the income gradient in mortality
Source: PLoS One. 2023 Jan 17;18(1):e0280272. doi: 10.1371/journal.pone.0280272 (PMC9844828; doi:10.1371/journal.pone.0280272)
Supplement: S1 Appendix — (DOCX) [file pone.0280272.s001.docx]

**Supporting Information: Appendix**

**Figures**

**Fig S0.** Map of the 90 *départements* considered in the article (classification that prevailed in 1967).


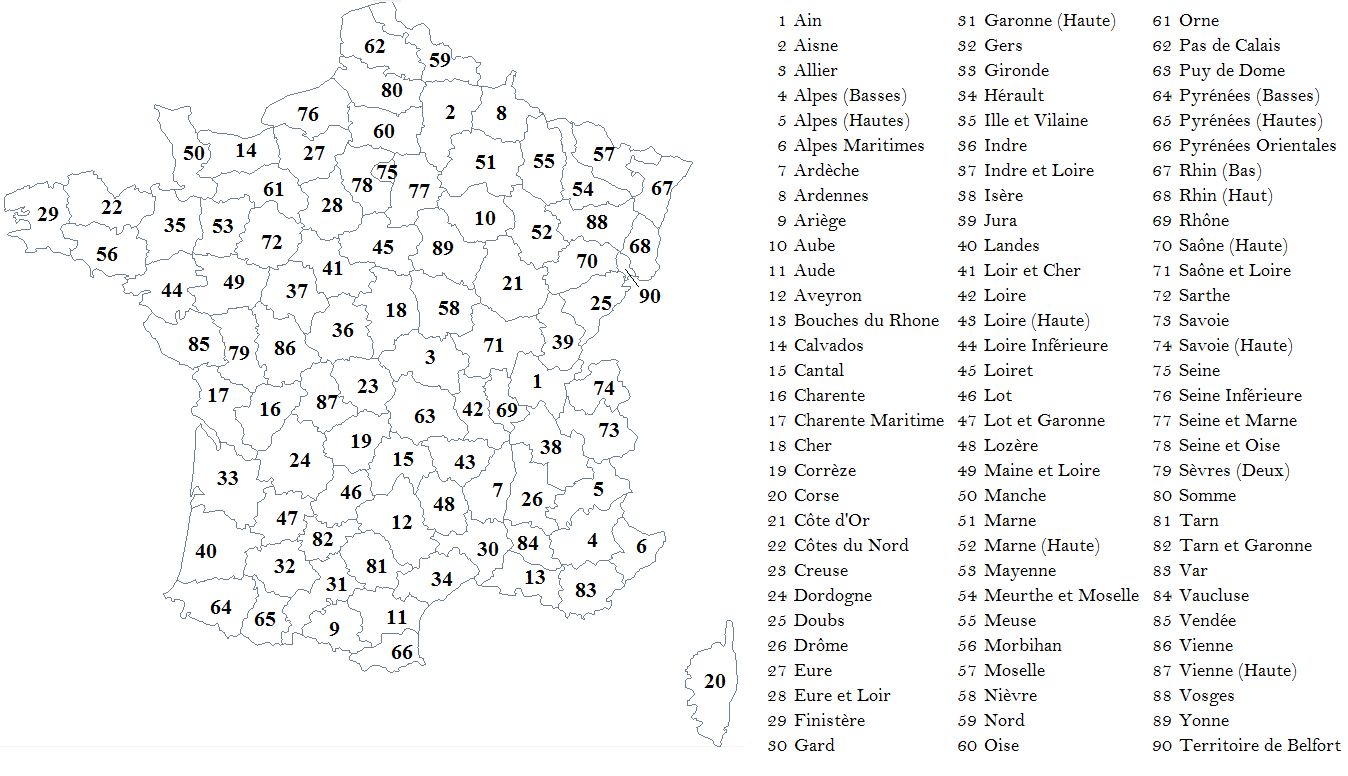


**Fig S1.** Death rates by inverted income per capita percentile, by age groups, men.

*Lines represent the linear trend that fits the annual death rates per 1,000 for 11 age groups and men across 20 income groups. A positive slope indicates that mortality decreases with income.*


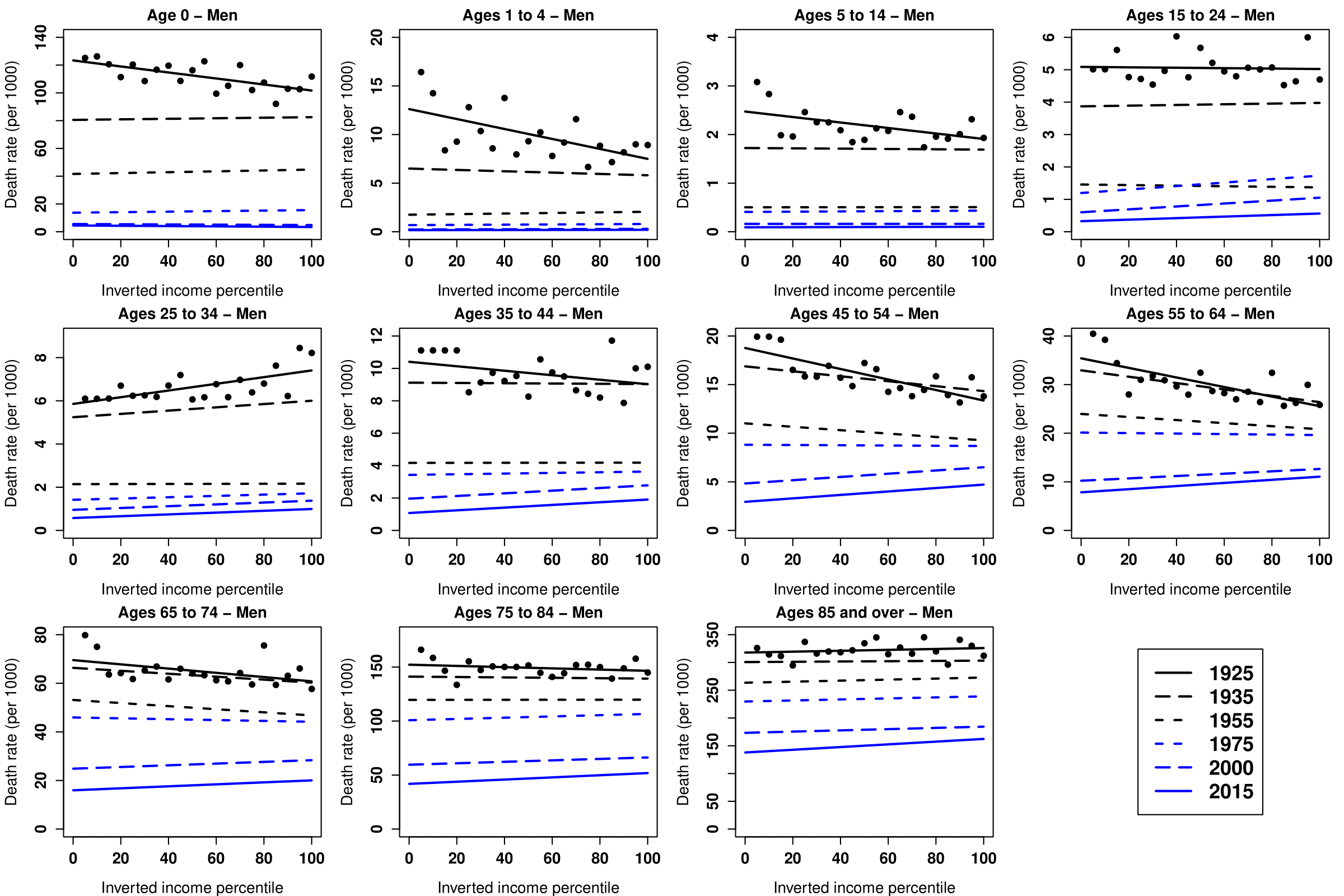


**Fig S2.** Death rates by inverted income per capita percentile, by age groups, women.

*Lines represent the linear trend that fits the annual death rates per 1,000 for 11 age groups and women across 20 income groups. A positive slope indicates that mortality decreases with income.*


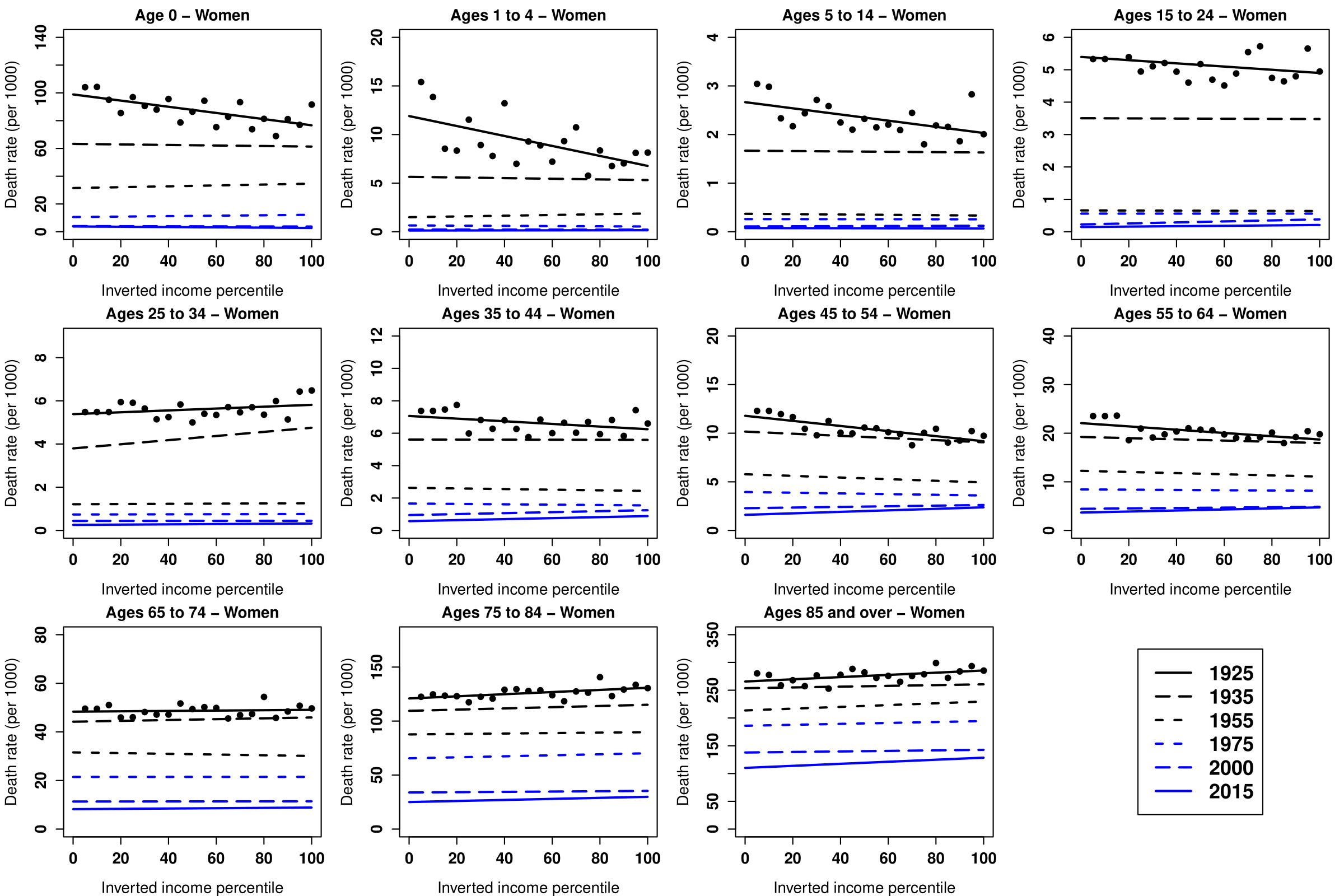


**Fig S3.** Death rates by income per capita.

*Lines represent the linear trend that fits the annual death rates per 1,000 for selected age groups and sex, by income per capita. A positive slope indicates that mortality increases with income.*


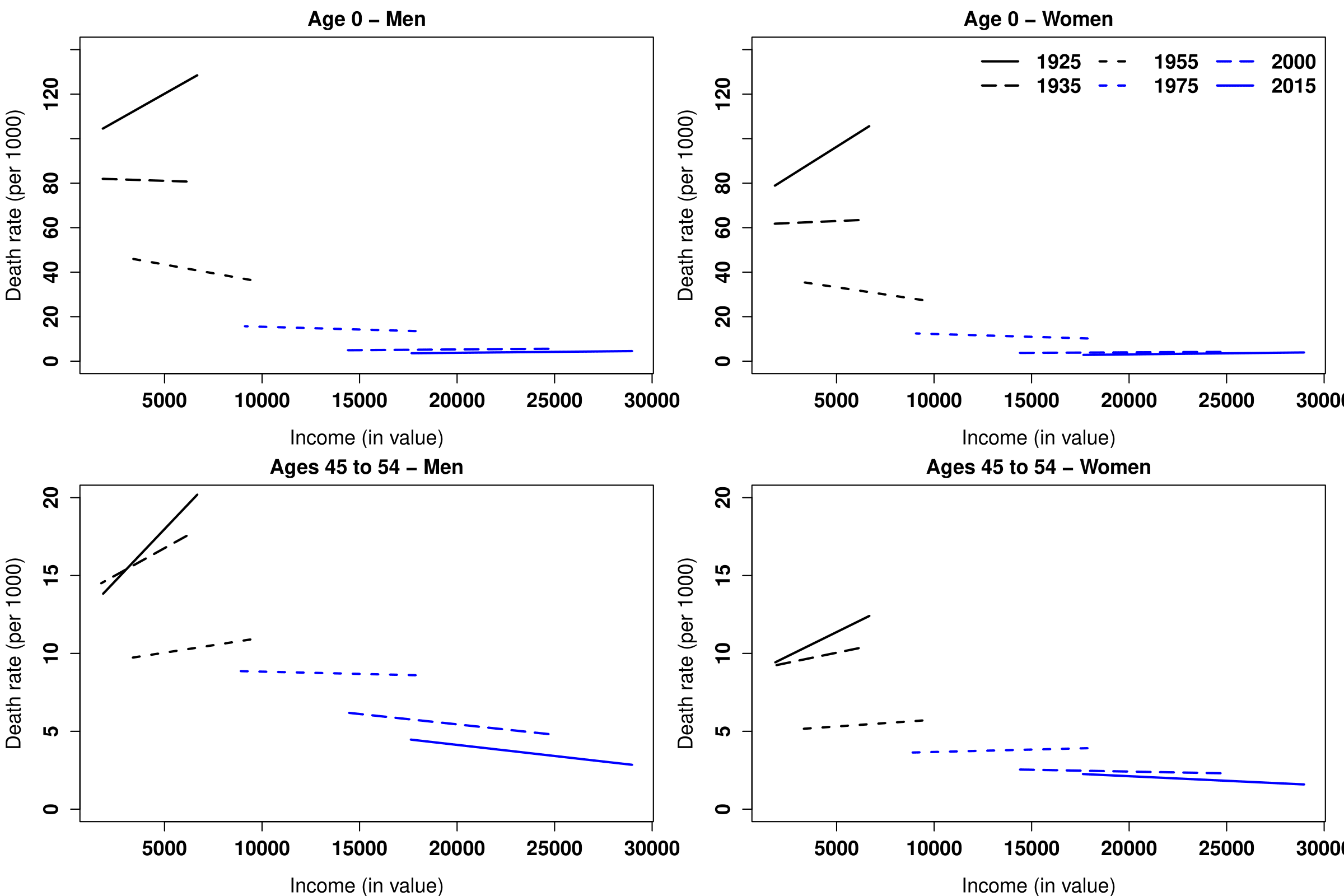


**Fig S4.** Income gradient in mortality for 11 age groups, men, 1922-2020.

*Plain lines plot the estimates of the linear trends across income groups; dotted lines are the 95% confidence intervals. Gradients are not represented for the period 1939-1945. Values of the gradients are provided in Table S3.*


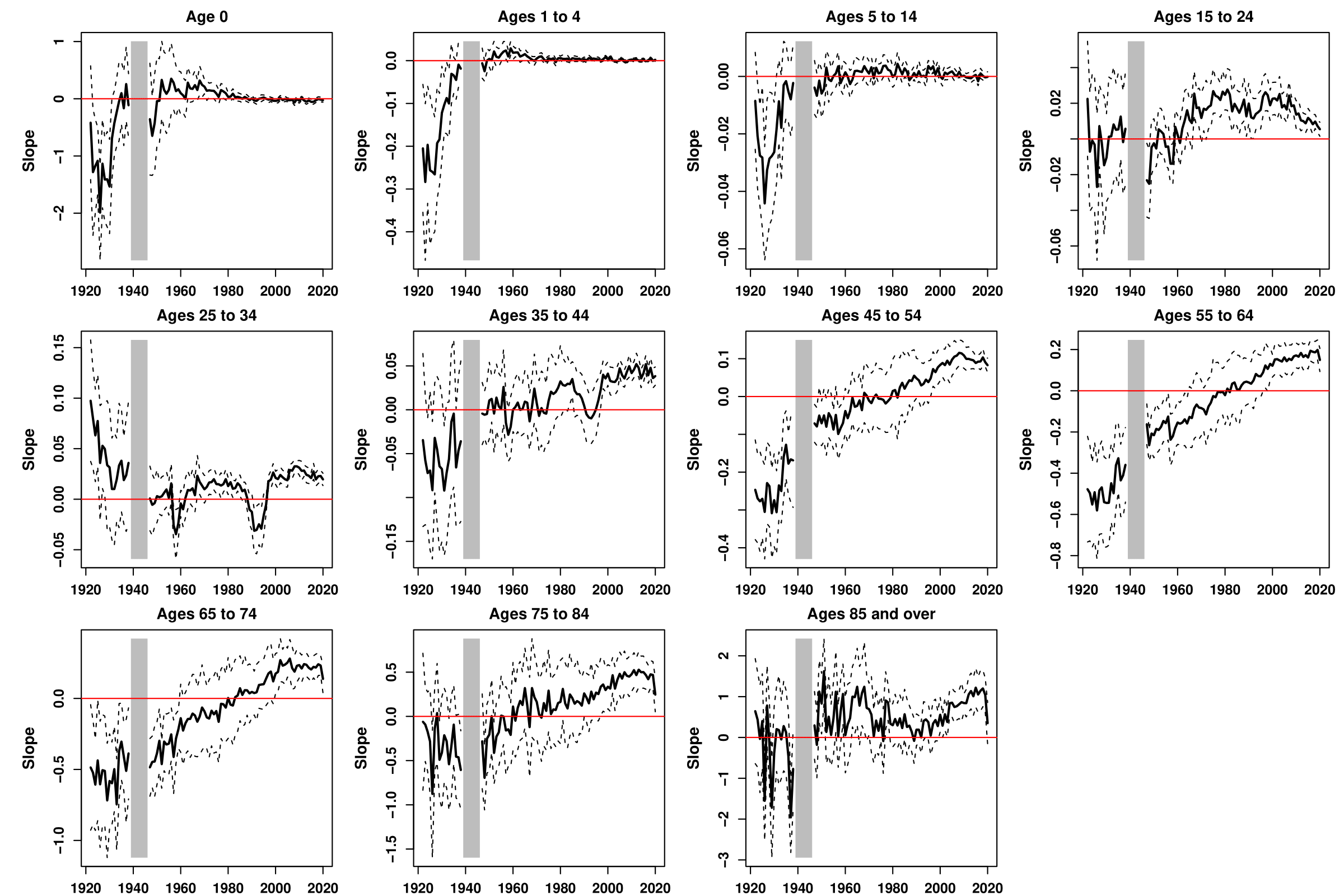


**Fig S5.** Income gradient in mortality for 11 age groups, women, 1922-2020.

*Plain lines plot the estimates of the linear trends across income groups; dotted lines are the 95% confidence intervals. Gradients are not represented for the period 1939-1945. Values of the gradients are provided in Table S3.*


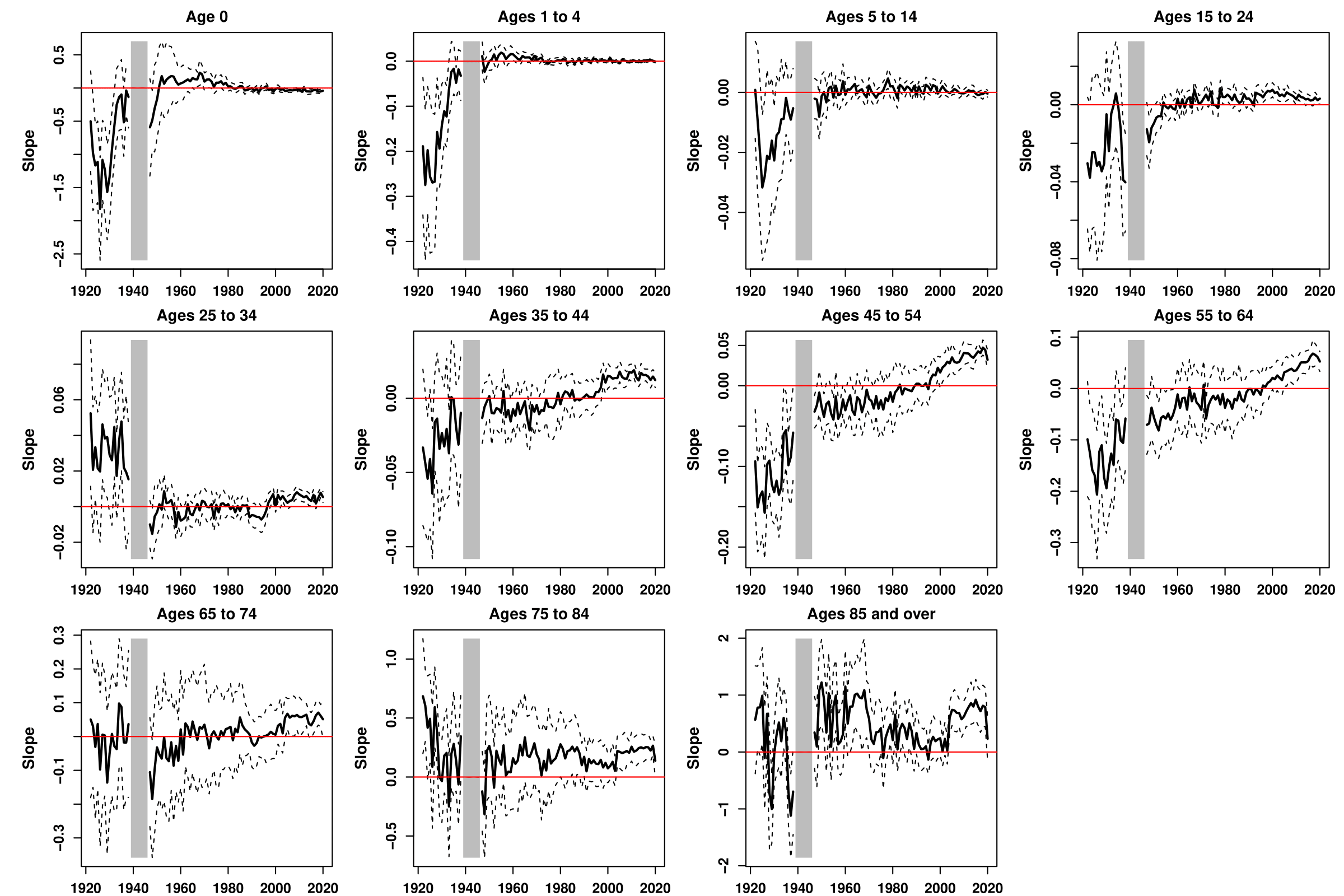


**Fig S6.** Income gradient in mortality for 6 age groups, 1968-2020, with the new classification of *départements* of the Paris region

*Plain lines plot the estimates of the linear trends across income groups; dotted lines are the 95% confidence intervals.*

**
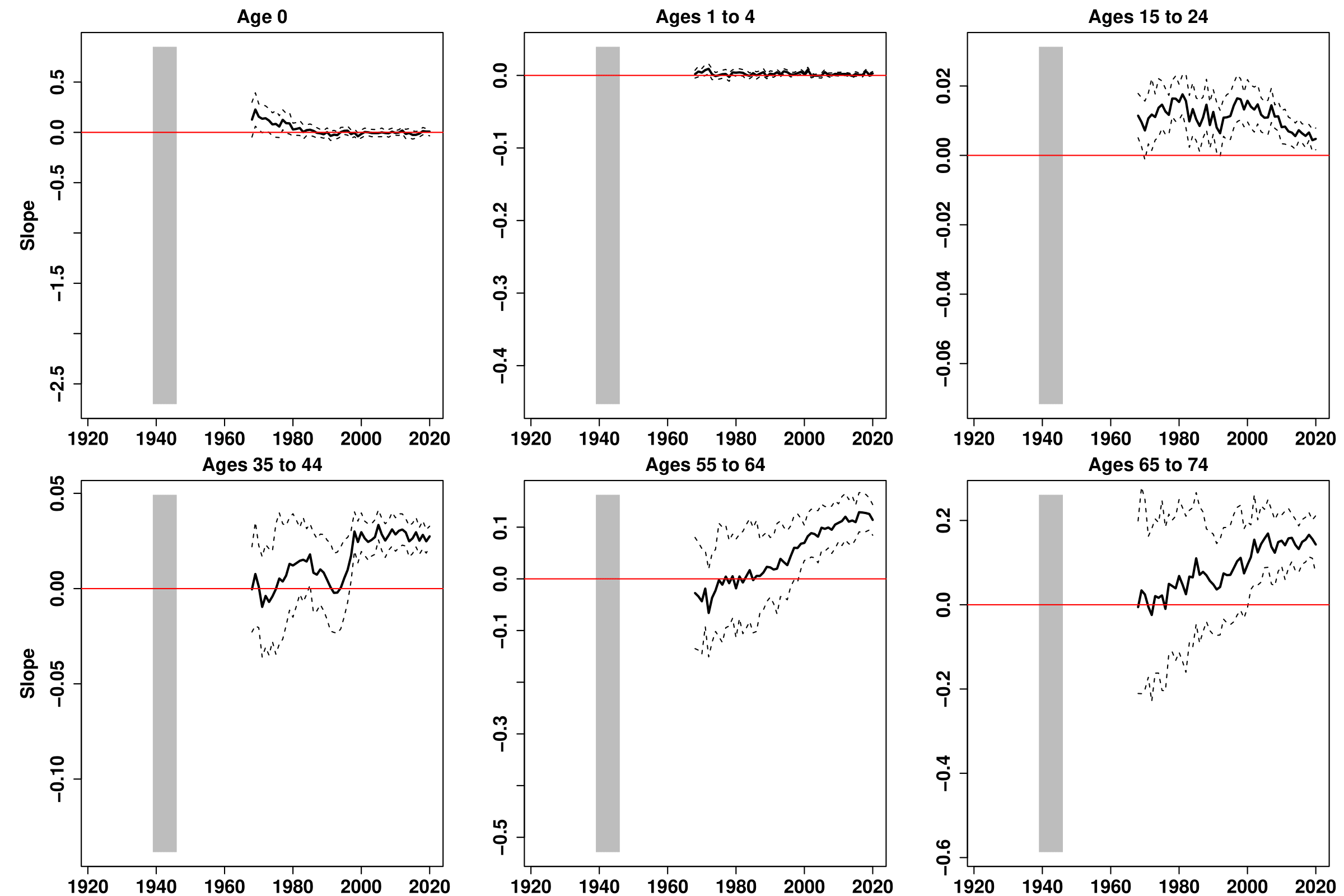
**

**Fig S7.** Income gradient in mortality for 4 mortality milestones, men, 1922-2020.

*Plain curves plot the estimates of the linear trends across income groups; dotted lines are the 95% confidence intervals. Red dots indicate dates at which the slope is significantly positive while blue dotes indicate those with significantly negative slopes. Gradients are not represented for the period 1939-1945. Values of the gradients are provided in Table S4.*


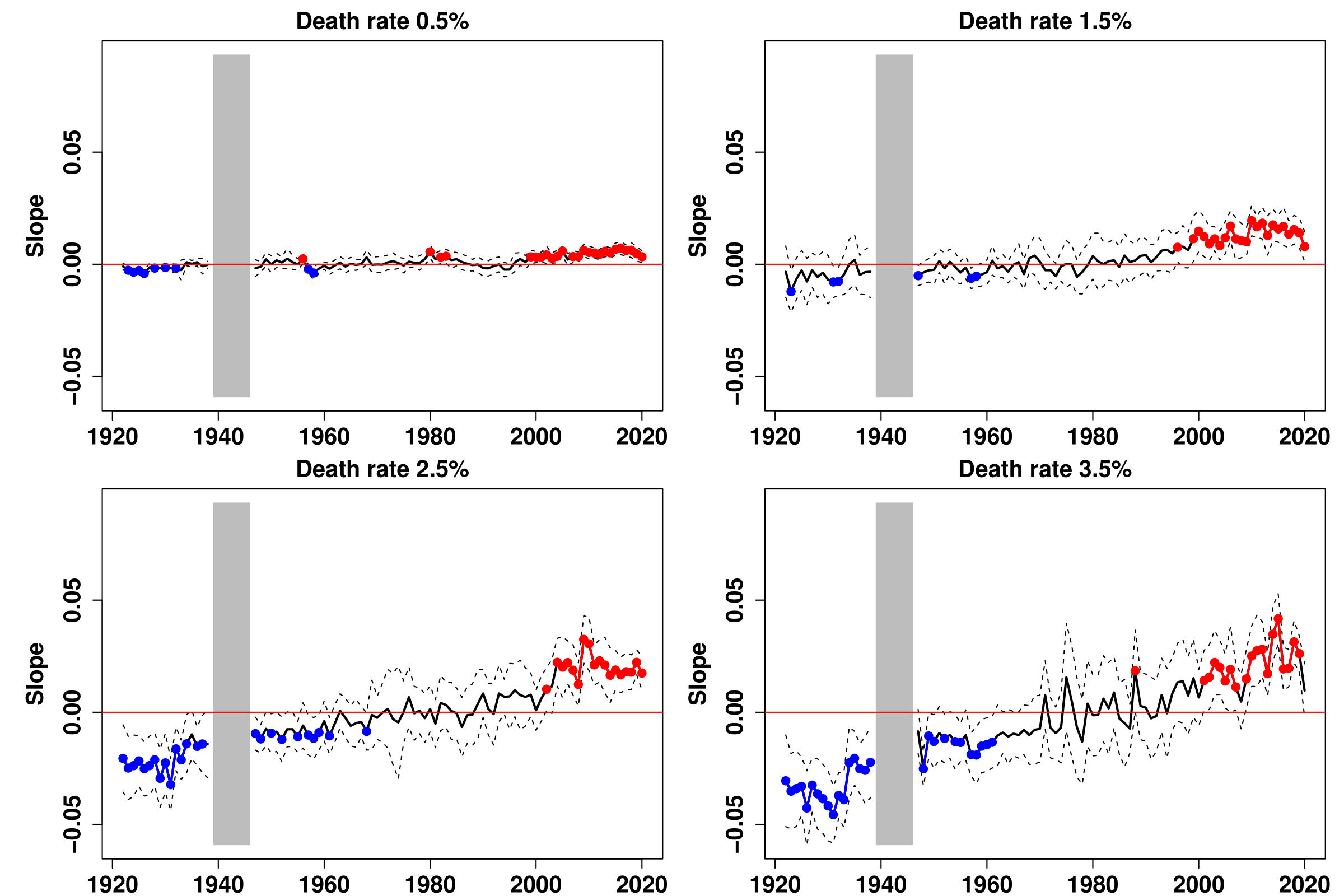


**Fig S8.** Income gradient in mortality for 4 mortality milestones, women, 1922-2020.

*Plain curves plot the estimates of the linear trends across income groups; dotted lines are the 95% confidence intervals. Red dots indicate dates at which the slope is significantly positive, while blue dots indicate those with significantly negative slopes. Gradients are not represented for the period 1939-1945. Values of the gradients are provided in Table S4.*


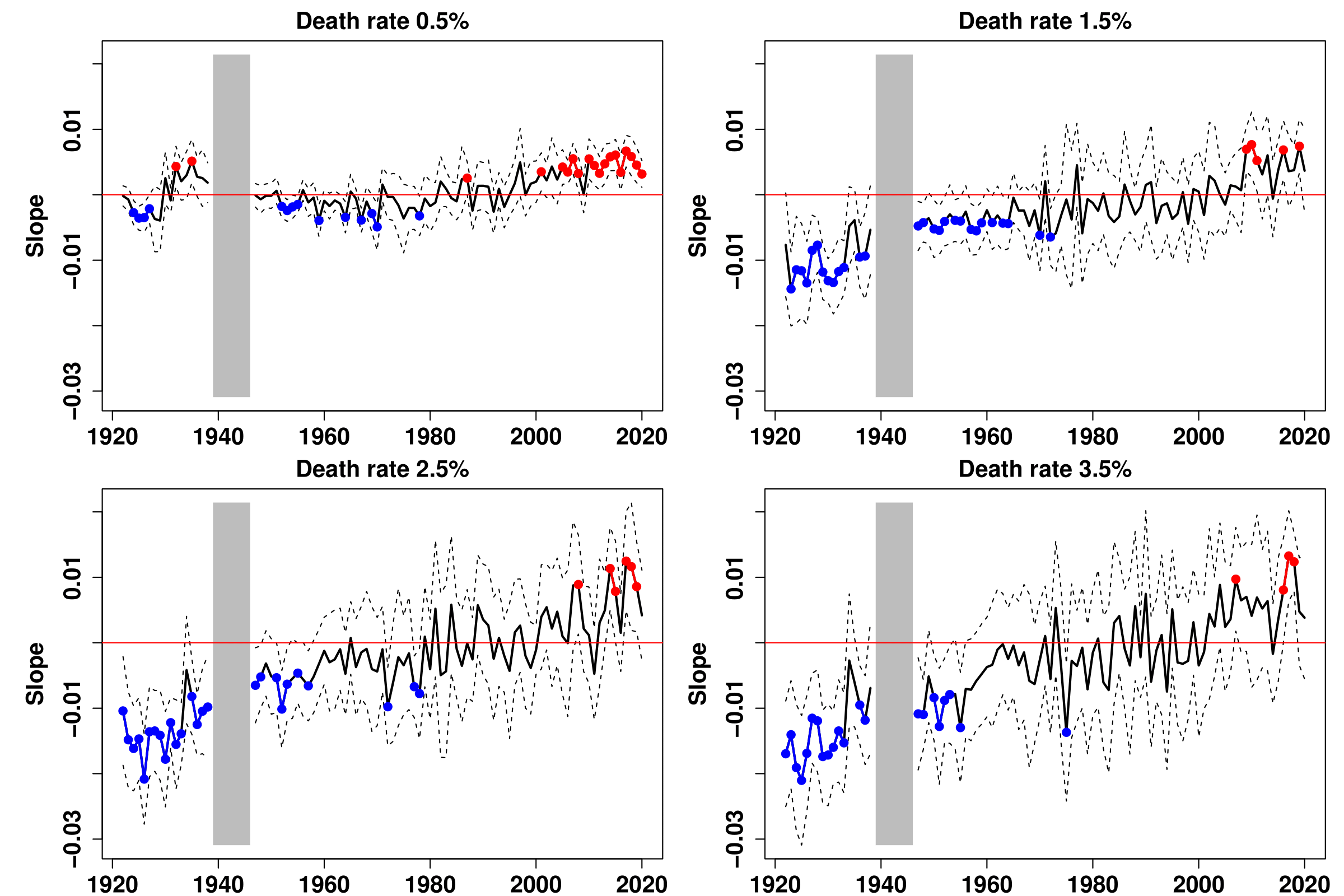


**Fig S9.** Income gradient in mortality for 4 mortality milestones without Bouches-du-Rhône, Rhône and Seine, 1922-2020.

*Plain curves plot the estimates of the linear trends across income groups computed without the highest densely populated regions (Bouches-du-Rhône, Rhône and Seine); dotted lines are the 95% confidence intervals. Red dots indicate dates at which the slope is significantly positive, while blue dots indicate those with significantly negative slopes. Gradients are not represented for the period 1939-1945.*


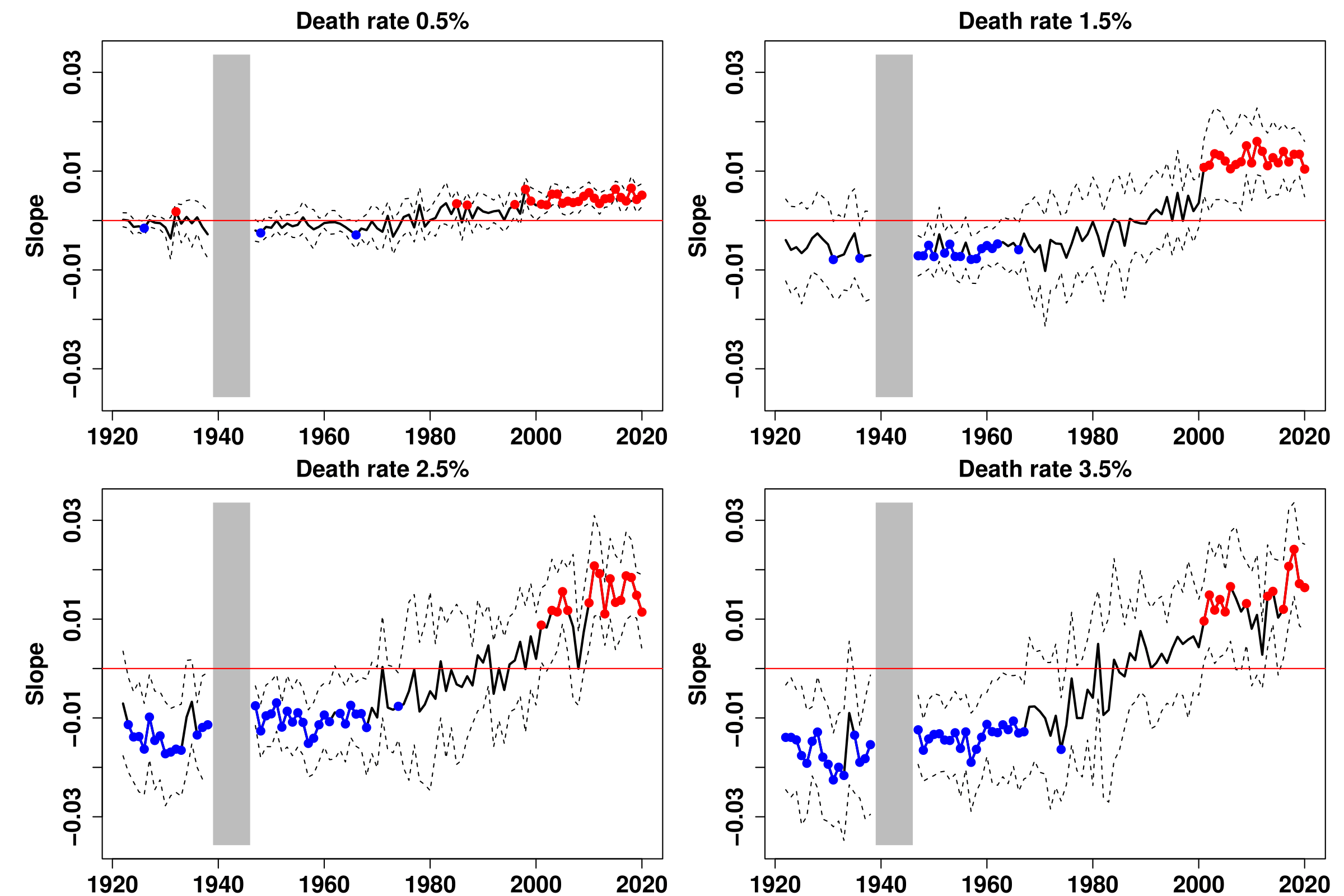


**Fig S10.** Income gradient in mortality for 6 age groups, 1968-2020 keeping constant the classification that prevailed in 1922.

*Plain lines plot the estimates of the linear trends across income groups; dotted lines are the 95% confidence intervals. Gradients are not represented for the period 1939-1945.*

**
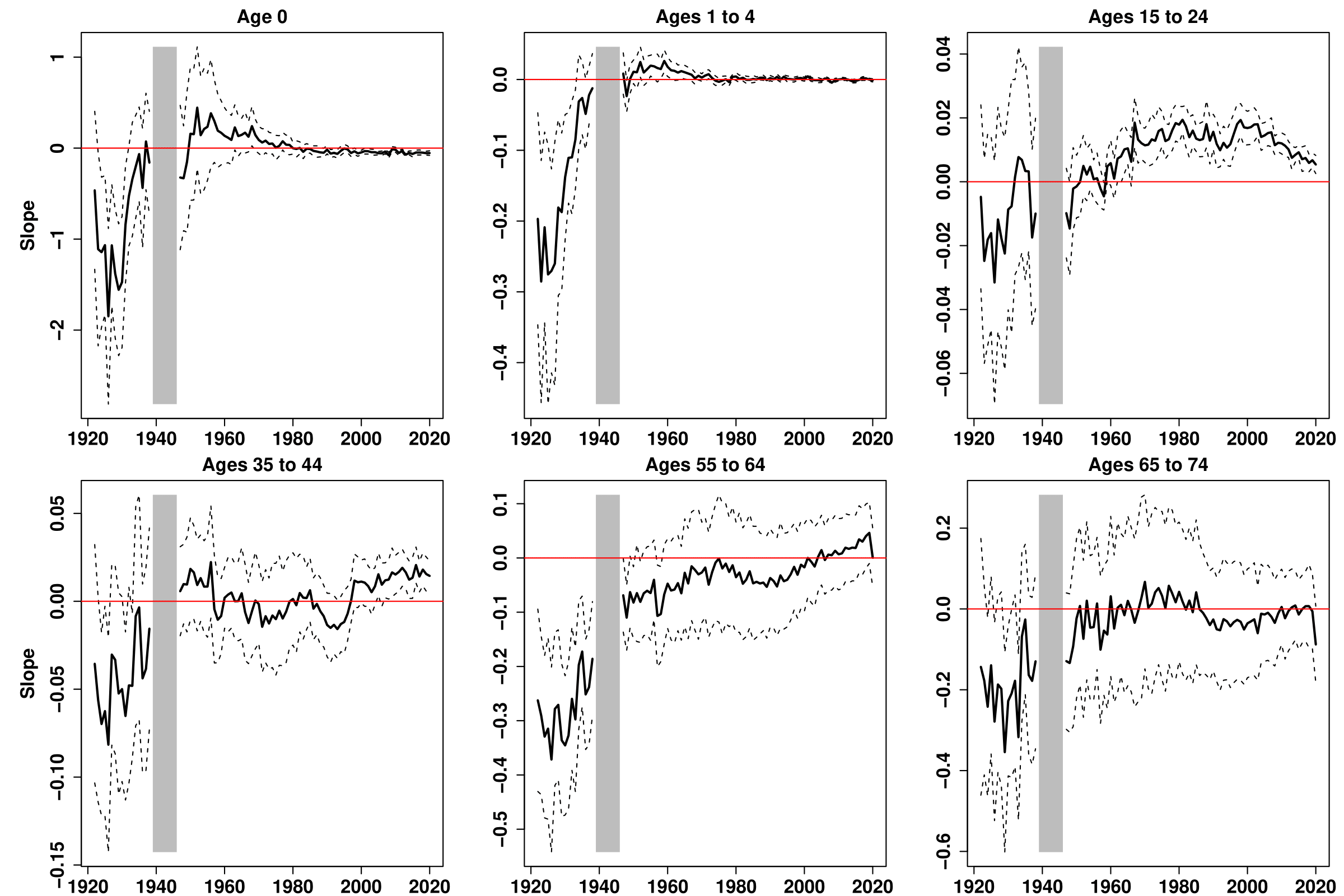
**

**Fig S11.** Income gradient in mortality for 6 age groups, 1968-2020 keeping constant the classification that prevailed in 2020.

*Plain lines plot the estimates of the linear trends across income groups; dotted lines are the 95% confidence intervals. Gradients are not represented for the period 1939-1945.*

**
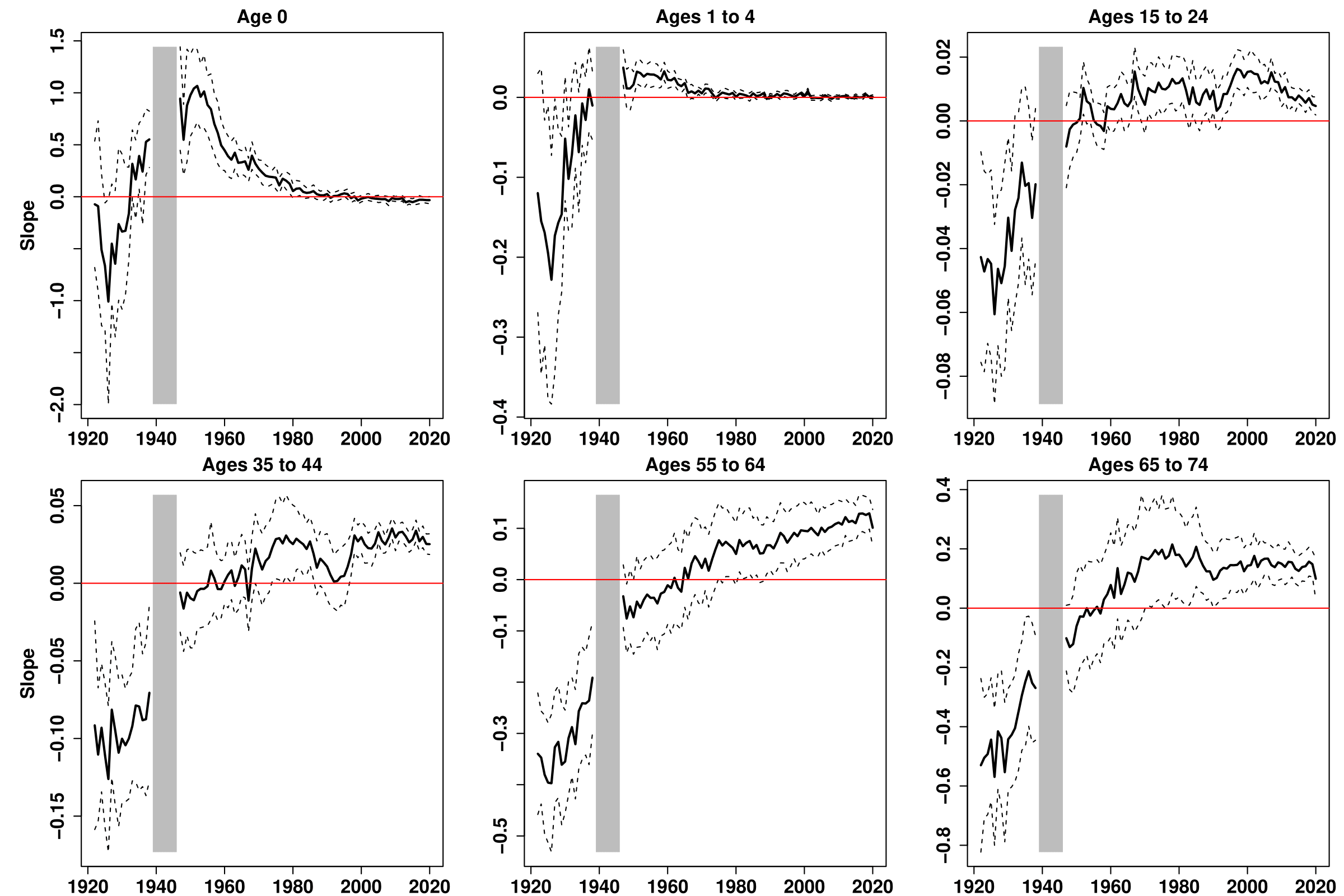
**

**Fig S12.** Density gradient in mortality for 6 age groups, 1922-2020.

*Plain curves plot the estimates of the linear trends across income groups; dotted lines are the 95% confidence intervals. Gradients are not represented for the period 1939-1945.*


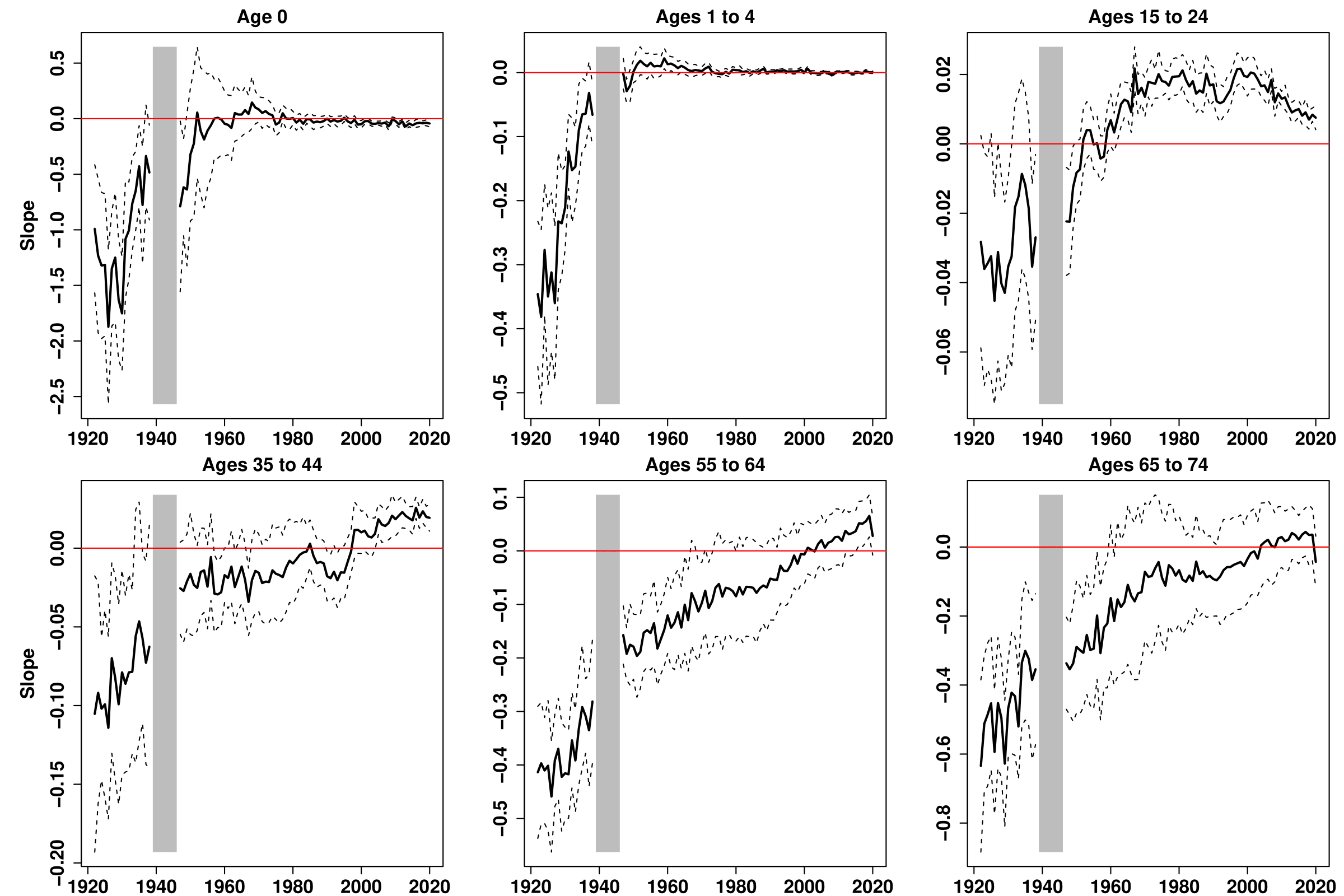


**Tables**

In the tables below, we provide some additional information that complement the figures. We selected 6 years (1925, 1935, 1955, 1975, 2000 and 2015) that are representative of the evolution. Values of all other years are available upon request.

**Table S1.** Mean fiscal income per capita for richest and poorest ventiles.

|  |  |  |  |  |  |  |
| --- | --- | --- | --- | --- | --- | --- |
| Ventile | Mean Income per capita | | | | | |
|  | 1925 | 1935 | 1955 | 1975 | 2000 | 2015 |
| Richest | 11.998 | 11.125 | 16.786 | 28.028 | 41.321 | 48.543 |
| Poorest | 2.633 | 2.588 | 4.496 | 13.319 | 22.699 | 28.796 |
|  |  |  |  |  |  |  |

*“Richest” refers to the ventile with the highest mean fiscal income per capita. “Poorest” is the ventile with the lowest mean fiscal income per capita. Values are in 2015 euros.*

**Table S2.** Death rates in richest and poorest ventiles.

|  |  |  |  |  |  |  |  |  |  |  |  |  |
| --- | --- | --- | --- | --- | --- | --- | --- | --- | --- | --- | --- | --- |
|  | 1925 | | 1935 | | 1955 | | 1975 | | 2000 | | 2015 | |
|  | Rich. | Poor. | Rich. | Poor. | Rich. | Poor. | Rich. | Poor. | Rich. | Poor. | Rich. | Poor. |
| Males |  |  |  |  |  |  |  |  |  |  |  |  |
| 0 | 131,34 | 111,88 | 84,26 | 97,08 | 33,6 | 46,77 | 15,16 | 15,73 | 4,64 | 3,8 | 3,89 | 3,21 |
| 1-4 | 16,37 | 9,68 | 6,83 | 6,21 | 1,45 | 1,93 | 0,74 | 0,75 | 0,46 | 0,52 | 0,26 | 0,25 |
| 5-14 | 3,07 | 2,11 | 1,78 | 1,71 | 0,44 | 0,54 | 0,46 | 0,47 | 0,13 | 0,18 | 0,08 | 0,09 |
| 15-24 | 5,01 | 5,29 | 3,66 | 4,15 | 1,38 | 1,31 | 1,13 | 1,59 | 0,49 | 0,86 | 0,32 | 0,56 |
| 25-34 | 6,1 | 8,39 | 5,36 | 6,61 | 1,89 | 1,96 | 1,5 | 1,5 | 0,92 | 1,28 | 0,6 | 0,98 |
| 35-44 | 11,11 | 10,5 | 9,74 | 10,07 | 3,79 | 3,43 | 3,33 | 2,79 | 2,19 | 2,82 | 1,15 | 1,92 |
| 45-54 | 19,92 | 14,53 | 17,43 | 14,6 | 10,48 | 8,15 | 8,34 | 6,93 | 5,33 | 7,04 | 3,11 | 4,66 |
| 55-64 | 40,47 | 27,48 | 33,72 | 26,89 | 22,63 | 19,31 | 18,42 | 16,97 | 10,39 | 14,37 | 7,97 | 11,75 |
| 65-74 | 79,78 | 59,74 | 68,9 | 59,03 | 51,13 | 45,39 | 43,1 | 40,68 | 23,05 | 30,77 | 16,08 | 22,94 |
| 75-84 | 167,42 | 154,49 | 144,51 | 140,7 | 118,64 | 118,71 | 93,65 | 102,88 | 56,03 | 70,59 | 38,37 | 55,53 |
| 85+ | 330,05 | 329,63 | 291,9 | 297,15 | 250,45 | 282,16 | 227,67 | 240,52 | 163,75 | 182,53 | 133,64 | 180,75 |
| Females |  |  |  |  |  |  |  |  |  |  |  |  |
| 0 | 107,81 | 88,32 | 65,03 | 70,97 | 25,43 | 36,12 | 10,44 | 10,32 | 3,95 | 3,3 | 3,51 | 2,52 |
| 1-4 | 15,38 | 9,18 | 6,04 | 6,56 | 1,23 | 1,98 | 0,67 | 0,45 | 0,46 | 0,22 | 0,27 | 0,24 |
| 5-14 | 3,04 | 2,36 | 1,63 | 1,7 | 0,33 | 0,33 | 0,29 | 0,24 | 0,11 | 0,13 | 0,09 | 0,09 |
| 15-24 | 5,33 | 5,5 | 3,38 | 3,99 | 0,6 | 0,64 | 0,54 | 0,6 | 0,24 | 0,34 | 0,14 | 0,15 |
| 25-34 | 5,48 | 6,64 | 3,84 | 5,49 | 1,19 | 1,17 | 0,79 | 0,72 | 0,45 | 0,41 | 0,26 | 0,32 |
| 35-44 | 7,38 | 7,53 | 5,77 | 6,76 | 2,53 | 2,3 | 1,68 | 1,34 | 1,01 | 1,24 | 0,61 | 0,79 |
| 45-54 | 12,3 | 9,88 | 10,19 | 10,6 | 5,48 | 4,85 | 3,94 | 3,17 | 2,43 | 2,75 | 1,68 | 2,49 |
| 55-64 | 23,53 | 19,48 | 18,7 | 19,83 | 11,47 | 10,81 | 7,93 | 7,35 | 4,77 | 5,42 | 3,82 | 4,92 |
| 65-74 | 49,57 | 49,1 | 42,5 | 47,65 | 29,44 | 30,2 | 19,56 | 20,79 | 11,03 | 12 | 8,37 | 9,44 |
| 75-84 | 122,39 | 132,17 | 103,32 | 120,55 | 83,28 | 90,41 | 61,42 | 67,69 | 31,63 | 36,81 | 23,83 | 30,13 |
| 85+ | 281,44 | 293,79 | 259,14 | 261,11 | 206,15 | 234,96 | 184,18 | 191,42 | 135,42 | 145,61 | 104,69 | 133,96 |
|  |  |  |  |  |  |  |  |  |  |  |  |  |

*“Richest” refers to the ventile with the highest mean fiscal income per capita. “Poorest” is the ventile with the lowest mean fiscal income per capita. Death rates are annual death rates per 1000.*

**Table S3.** Income gradient by sex and age groups for selected years.

|  |  |  |  |  |  |  |
| --- | --- | --- | --- | --- | --- | --- |
|  | 1925 | 1935 | 1955 | 1975 | 2000 | 2015 |
| Men |  |  |  |  |  |  |
| 0 | -1.0781* | 0.2489 | 0.3793 | 0.1039 | -0.0338 | -0.0498* |
| 1-4 | -0.2763* | -0.0311 | 0.0205* | 0.0058 | 0.0002 | -0.0001 |
| 5-14 | -0.0307* | -0.0006 | 0.0018 | 0.001 | -0.0005 | 0.0007 |
| 15-24 | -0.0071 | 0.002 | -0.002 | 0.0273* | 0.0231* | 0.0119* |
| 25-34 | 0.0722* | 0.0364 | 0.0044 | 0.0135* | 0.0171* | 0.0181* |
| 35-44 | -0.0563 | 0.0098 | 0.0073 | 0.0232 | 0.0363* | 0.0378* |
| 45-54 | -0.2435* | -0.1005* | -0.0666 | 0.0337 | 0.0955* | 0.0845* |
| 55-64 | -0.51* | -0.261* | -0.1019 | 0.0716 | 0.1671* | 0.1721* |
| 65-74 | -0.5635* | -0.2357 | -0.158 | 0.161 | 0.298* | 0.2679* |
| 75-84 | -0.2661 | 0.1819 | 0.3875 | 0.7007* | 0.5937* | 0.6289* |
| 85+ | 0.4533 | 0.0212 | 1.5387* | 0.9793* | 0.9177* | 1.5866* |
| Women |  |  |  |  |  |  |
| 0 | -1.1966* | 0.0688 | 0.3238 | 0.0864 | -0.0064 | -0.0394* |
| 1-4 | -0.2929* | -0.013 | 0.0252* | -0.0057 | -0.0035 | -0.0009 |
| 5-14 | -0.0373* | 0.0001 | -0.001 | -0.0013 | 0.0008 | 0.0004 |
| 15-24 | -0.0286 | 0.0037 | -0.0004 | 0.0002 | 0.0069* | 0.0022 |
| 25-34 | 0.0192 | 0.0499* | 0.0039 | -0.0004 | 0.0008 | 0.0031 |
| 35-44 | -0.0323 | 0.0071 | -0.0061 | -0.0023 | 0.0151* | 0.0146* |
| 45-54 | -0.125* | -0.0363 | -0.0332* | 0.0006 | 0.0214* | 0.0379* |
| 55-64 | -0.1644* | -0.0197 | -0.0335 | 0.0213 | 0.0316* | 0.0535* |
| 65-74 | 0.0311 | 0.1825* | 0.0024 | 0.0901 | 0.0409 | 0.046* |
| 75-84 | 0.5691* | 0.5278* | 0.3075 | 0.4598* | 0.1805* | 0.2712* |
| 85+ | 1.0769* | 0.626 | 1.174* | 0.7536* | 0.4294* | 1.1404* |
|  |  |  |  |  |  |  |

*Income gradients are estimates of the linear trends across income groups. Values with (*) are significant at the 5% threshold.*

**Table S4.** Income gradient by sex and mortality milestones, for selected years.

|  |  |  |  |  |  |  |
| --- | --- | --- | --- | --- | --- | --- |
|  | 1925 | 1935 | 1955 | 1975 | 2000 | 2015 |
| Men |  |  |  |  |  |  |
| 0.5 | -0.0013 | 0.0008 | 0.001 | 0.0004 | 0.0031 | 0.0066* |
| 1.5 | -0.0041 | 0.001 | -0.0027 | 0.0039 | 0.019* | 0.0162* |
| 2.5 | -0.0212* | -0.0097* | -0.0084 | 0.0041 | 0.0043 | 0.0225* |
| 3.5 | -0.032* | -0.0187* | -0.0099 | 0.0213 | 0.0141* | 0.0455* |
| Women |  |  |  |  |  |  |
| 0.5 | -0.0017 | 0.005* | -0.0004 | -0.0019 | 0.0025 | 0.0061* |
| 1.5 | -0.0115* | -0.0029 | -0.0027 | 0.0001 | 0.0029 | 0.0042 |
| 2.5 | -0.0145* | -0.0061* | -0.0022 | 0.0004 | 0.001 | 0.0088 |
| 3.5 | -0.0205* | -0.0035 | -0.0098 | -0.0083 | 0.001 | 0.0059 |
|  |  |  |  |  |  |  |

*Income gradients are estimates of the linear trends across income groups. Values with (*) are significant at the 5% threshold. Mortality milestones are in %.*
